# Genetic Interactions of *Arabidopsis thaliana Damaged DNA Binding Protein 1B* (*DDB1B*) With *DDB1A*, *DET1*, and *COP1*

**DOI:** 10.1534/g3.112.005249

**Published:** 2013-03-01

**Authors:** Ashwin L. Ganpudi, Dana F. Schroeder

**Affiliations:** Department of Biological Sciences, University of Manitoba, Winnipeg, MB, Canada R3T 2N2

**Keywords:** DDB1B, DDB1A, DET1, COP1, light

## Abstract

Damaged DNA Binding protein 1 (DDB1)–CULLIN4 E3 ubiquitin ligase complexes have been implicated in diverse biological processes in a range of organisms. *Arabidopsis thaliana* encodes two homologs of DDB1, DDB1A, and DDB1B. In this study we use a viable partial loss of function allele of *DDB1B*, *ddb1b-2*, to examine genetic interactions with *DDB1A*, *DET1* and *COP1*. Although the *ddb1b-2 ddb1a* double mutant is lethal, *ddb1a ddb1b-2/+* and *ddb1b-2 ddb1a/+* heterozygotes exhibit few developmental phenotypes but do exhibit decreased tolerance of ultraviolet light. In addition, germination in *ddb1a* and *ddb1a ddb1b-2/+* was found to be sensitive to salt and mannitol, and both *DDB1* single mutants as well as the heterozygotes exhibited heat sensitivity. DE-ETIOLATED1 (DET1) and CONSTITUTIVE PHOTOMORPHOGENIC1 (COP1) are negative regulators of light development which interact with DDB1-CUL4 complexes. Although *ddb1a* strongly enhances *det1* phenotypes in both dark- and light-grown seedlings, *ddb1b-2* weakly enhanced the *det1* short hypocotyl phenotype in the dark, as well as enhancing anthocyanin levels and suppressing the *det1* low chlorophyll phenotype in light-grown seedlings. In adults, *ddb1a* suppresses *det1* early flowering and enhances the *det1* dwarf phenotype. A similar trend was observed in *ddb1b-2 det1* double mutants, although the effects were smaller in magnitude. In *cop1* mutants, *ddb1b-2* enhanced the *cop1-4* short hypocotyl phenotype in dark and light, enhanced anthocyanin levels in *cop1-1* in the light, but had no effect in adults. Thus the requirement for *DDB1B* varies in the course of development, from *COP1*-specific effects in hypocotyls to *DET1*-specific in adults.

Light, an essential environmental cue, has profound effects on all stages of plant growth and development. Under dark conditions, seedlings follow a skotomorphogenic (or etiolated) growth pattern (elongated hypocotyls and closed unexpanded cotyledons protected by an apical hook). In contrast, upon perceiving light, seedlings switch to a photomorphogenic (or de-etiolated) growth pattern (short hypocotyls and open expanded cotyledons with active chloroplast differentiation). This transition from etiolation to de-etiolation is controlled by the *COP/DET/FUS* genes. All of the pleiotropic *Arabidopsis thaliana cop/det/fus* mutants display a de-etiolated (*det*) or constitutively photomorphogenic (*cop*) phenotype in the absence of light, with increased anthocyanin accumulation, partial chloroplast development and differential expression of hundreds of light-regulated genes (Chen and Chory 2011; Lau and Deng 2012).

The *COP/DET/FUS* genes have been cloned and shown to be involved in protein degradation. Six of the *COP/DET/FUS* family genes encode components of the COP9 signalosome. The COP9 signalosome exhibits high homology to the 19S lid subcomplex of the 26S proteosome and regulates CULLIN-based E3 ubiquitin ligases via deconjugating/conjugating RUB / NEDD8 (Wei *et al.* 2008). COP1 is a RING-finger protein with a zinc finger motif at the N terminus, followed by a coiled-coil domain and seven WD40 repeats at the C terminus. Cellular localization of COP1 is light-regulated. Several positive regulators of photomorphogenesis, such as HY5, HYH, LAF1, and HFR1, as well as the photoreceptor Phytochrome A are targeted for degradation via interaction with the COP1 WD40 domain (Yi and Deng 2005). DET1, a 62-kDa nuclear localized protein, associates with nonacetylated core histones (Benvenuto *et al.* 2002), has been implicated as a transcriptional repressor (Lau *et al.* 2011), and exhibits biochemical and genetic interactions with DDB1A. Arabidopsis encodes two homologs of DDB1—DDB1A and DDB1B (Schroeder *et al.* 2002). DET1 and DDB1A interact with COP10 to form the CDD complex, which in turn interacts with CULLIN4 (Yanagawa *et al.* 2004; Bernhardt *et al.* 2006; Chen *et al.* 2006). Interestingly, COP1 also interacts biochemically with DDB1A and DDB1B as well as CULLIN4 (Chen *et al.* 2010).

DDB1 and DDB2 are core components of the ultraviolet (UV)-damaged DNA-binding protein complex (DDB) initially identified in human cells. The primary UV-induced DNA lesions include cyclobutane pyrimidine dimers and 6-4 pyrimidine-pyrimidone photoproducts (Kunz *et al.* 2006). To counteract this damage, plants employ specific mechanisms: photoreactivation, catalyzed by the blue light-dependent photolyase class of enzymes, and the light-independent nucleotide excision repair (NER) pathway. The dark repair pathway, NER, has specific repair subpathways for transcriptionally active (transcription coupled repair [TC-NER]) or silent (global genomic repair [GG-NER]) DNA regions. Both TC-NER and GG-NER exhibit different damage recognition strategies followed by a common repair pathway. In human GG-NER, lesion recognition is mediated by the CUL4-DDB1^-DDB2^ complex followed by XPC-HR23B-CEN2 recruitment. In human TC-NER, CUL4-DDB1^-CSA^ recognizes the stalled RNA POL II bound to CSB. Thus both subpathways of NER are regulated by a CUL4-DDB1 complex interacting with specific recognition substrates: DDB2 (in GG-NER) and CSA (Cockayne Syndrome A; in TC-NER). Following recognition, both mechanisms employ a common repair pathway (Ganpudi and Schroeder 2011).

DDB1 is 127 kDa and composed of three β propeller domains (BPA, BPB, and BPC). BPB interacts with CUL4, and the clam-shaped BPA−BPC pocket mediates interactions with a large number of proteins containing WD40 domains, referred to as DCAF proteins (*i.e.*, DDB1-CUL4−associated factor) or DWD proteins (*i.e.*, DDB1 binding WD40 proteins) (Lee and Zhou 2007; Biedermann and Hellmann 2011). The Arabidopsis genome encodes approximately 230 WD40 proteins but only a fraction of them (approximately 86 proteins) have one or more WDxR motifs within the WD40 domain capable of interacting with DDB1 (Lee *et al.* 2008). Arabidopsis DDB1A and DDB1B are 91% identical and 97% similar at the amino acid level (Schroeder *et al.* 2002; Bernhardt *et al.* 2010). The differences between DDB1A and DDB1B are distributed fairly evenly throughout the proteins, with the exception of a region between amino acids 729−766, which decreases to 59% identity and 78% similarity. This region maps to the loop 3b-3c region of DDB1 BPC, which is somewhat variable, and in fact has an extra loop in animal DDB1s. This region is on the bottom of BPC and is not directly involved in interactions yet mapped (Li *et al.* 2006). Consistent with this, many proteins, including CUL4, DDB2, DET1, COP1, SPA1-SPA4, DWA1-DWA3, FY, PRL1, TRIP-1, VIP3, MSI3, and MSI4/FVE, have been found to interact with both DDB1A and DDB1B in yeast two-hybrid and/or coimmunoprecipitation experiments (Bernhardt *et al.* 2006; Lee *et al.* 2008; Bernhardt *et al.* 2010; Chen *et al.* 2010; Lee *et al.* 2010; Dumbliauskas *et al.* 2011; Lee *et al.* 2011; Pazhouhandeh *et al.* 2011).

Duplication of the *DDB1* gene appears to be specific for the Brassicaceae, because clear *DDB1A* and *DDB1B* homologs exist in *Brassica rapa*, *Capsella rubella*, and *Arabidopsis lyrata* in addition to *Arabidopsis thaliana* (www.phytozome.net). Evolution of these genes appears constrained because the Ka/Ks ratio (0.04/0.57) is well below one (Wagner 2002; Singh *et al.* 2010). *DDB1A* and *DDB1B* are expressed throughout Arabidopsis development with *DDB1A* levels on average twofold greater than those of *DDB1B* (Al Khateeb and Schroeder 2007; Bernhardt *et al.* 2010). Null alleles of *DDB1A* do not exhibit obvious developmental phenotypes while null alleles of *DDB1B* appear lethal (Schroeder *et al.* 2002). Up-regulated levels of both *DDB1A* and *DDB1B* mRNA are observed after UV irradiation, and mild-to-severe UV sensitivity was observed in *ddb1a* and *ddb2* mutants and overexpression of *DDB1A* and *DDB2* confers increased UV resistance (Koga *et al.* 2006; Molinier *et al.* 2008; Al Khateeb and Schroeder 2009). In this study we examine the role of *DDB1B* by analyzing the genetic interactions of a partial loss of function *DDB1B* allele with *DDB1A*, *DET1*, and *COP1*.

## Materials and Methods

### Plant materials and growth conditions

All lines in this study were in the Col background of *Arabidopsis thaliana*. *det1-1*, *ddb1a*, and *det1 ddb1a* were as previously described (Chory *et al.* 1989; Schroeder *et al.* 2002). Strong and weak alleles of *cop1*, *cop1-1* and *cop1-4* respectively, were kindly provided by XW Deng (Yale University). The *ddb1b-2* allele (SALK_061944) was obtained from the Arabidopsis Stock Center (Alonso *et al.* 2003). Various double mutant combinations were generated using standard protocols (Weigel and Glazebrook 2002). *ddb1a* genotyping was as described in Al Khateeb and Schroeder (2007). For *ddb1b-2*, the T-DNA insertion was detected using LB2 (TTGGGTGATGGTTCACGTAGTGGGCCATCC) and UV2.21 (CAGAGAAGGAAACCAAGGGAGC) whereas wild-type *DDB1B* was detected using UV2.21 and *DDB1B* 3′UTR (AGGGGAAGAGGAGAGCTTGGA). Because *ddb1a ddb1b-2* is embryonic lethal, these lines were maintained as *ddb1b-2 ddb1a/+* and *ddb1a ddb1b-2/+* heterozygotes. Seeds were sterilized and plated on Linsmaier and Skoog media (Caisson) supplemented with either 2% sucrose (*det1* and *cop1* experiments) or 0.6% sucrose (*ddb1b-2 ddb1a* experiments) and 0.8% Phytoblend (Caisson). After 2 d of stratification at 4°, plates were transferred to a growth chamber (20° with 50% relative humidity). Light was provided by fluorescent bulbs (100 µM photons m^-2^ sec^-1^). Short-day conditions correspond to 10-hr light:14-hr dark relative to long-day conditions, which correspond to 16-hr light:8-hr dark. For adult growth, 14-d-old seedlings were transplanted to Sunshine Mix Number 1 (SunGro, Bellevue, WA).

### RNA extraction and reverse transcription polymerase chain reaction (RT-PCR)

Total RNA was extracted from 7-d-old seedlings using a RNeasy plant minikit (QIAGEN) according to the manufacturer’s instructions including a DNase step. Quantity of extracted RNA was measured by spectroscopic analysis based on UV absorbance. cDNA synthesis and amplification was a one step process using an Access RT-PCR kit (Promega). Semiquantitative RT-PCR was performed at 45° for 45 min followed by PCR [5 min 94°, (30 sec 94°, 50 sec 53°, 90 sec 72°) for a gene-specific number of cycles, then 7 min 72°]. *Actin* was used as the loading control. PCR products were separated on 1% (w/v) agarose ethidium bromide-stained gels and band intensities were analyzed using Image Lab 3.0 (Biorad). The following primers were used: *DDB1B* 2.21 (P3) CAGAGAAGGAAACCAAGGGAG, 2.27 (P4) CACACAATGAAACTCTTATTAA, 22 cycles; for *DDB1A* 10XL (P7) TAAAGAAGTTAGTCATATGTGCCCT, 1.4 (P8) GCAACCTCCCATCTTCGACTATAAATACTA, 20 cycles; total *DDB1A + DDB1B* X.12 (C1) GGAGCTGTTTATTCTCTCAA, 2.20 (C2) TGCACAACTCTTCCACTTGAAC, 22 cycles; *CAB2* CAGCAGGTGGGCCATGCTCG, GCCTCTACAACGGAGTGAACCCAA, 21 cycles; and *Actin* CTGGAACAAGACTTCTGGGC, GGTGATGAAGCACAATCCAAG, 24 cycles.

### Seedling analysis

For hypocotyl length and cotyledon width assays, 7-d-old seedlings grown in either long-day or dark conditions (after an initial 6-hr light treatment) were scanned and analyzed using NIH Image software. For chlorophyll content analysis 7-d-old seedlings were extracted with 80% acetone overnight, A_645_ and A_663_ was determined in a spectrophotometer (model 2100 pro Ultrospec) and chlorophyll content calculated according to the MacKinney method (Mackinney 1941). Anthocyanin content was determined using standard protocol as described in Fankhauser and Casal (2004). Pigment analysis experiments were repeated at least three times with two replicates per line in each experiment.

### Adult growth parameters

14-d-old seedlings were transplanted to soil. General growth parameters such as flowering time (number of days until bud emergence and number of rosette and cauline leaves), rosette diameter (at 4 wk) and plant height, apical dominance, and silique length (at approximately 6 wk) were determined.

### UV tolerance assays

#### Shoot assays:

Twenty-one-day-old plants were irradiated with 450 J m^-2^ UV-C light (254 nm) using a UV lamp (Model XX-15S; UVP, Upland, CA) with a flux rate of 1.6 mW cm^-2^. After irradiation, plants were dark incubated for 3 d then transferred to standard growth conditions where percentage sensitivity was assessed by leaf yellowing and necrosis.

#### Root assays:

Seeds were grown on vertically oriented plates for 3 d under the same long-day growth conditions as mentioned previously. For light assays, plates were irradiated with 600 J m^-2^ UV-C, rotated 90° and incubated vertically under long-day conditions for 2 d. Fresh root growth (starting from the bending point) was measured using NIH Image. For dark assays, plates were UV-C irradiated with 1500 J m^-2^, similarly rotated and incubated under dark conditions for 3 d. New growth was detected by bending assay and measured using NIH Image.

### Seedling abiotic stress experiments

For germination assays (horizontally aligned) and for root length assays (vertically aligned) plates supplemented with either 100 mM NaCl or 200 mM Mannitol were used. Germination was scored 3 d after transfer to long-day conditions and root growth was measured 7 d after transfer to long-day conditions.

For heat assays, seedlings plated on equal volumes of growth medium were dark-grown for 4 d, followed by heat treatment (45°) for 4 hr. The hypocotyl length was measured after an additional 4 d of dark growth posttreatment.

### Statistical analysis

All experiments were repeated at least three times. Data were compared by Student’s *t*-test and probabilities of 0.05 or less considered statistically significant.

## Results

### Interactions between *ddb1b* and *ddb1a*

**Figure 1:**
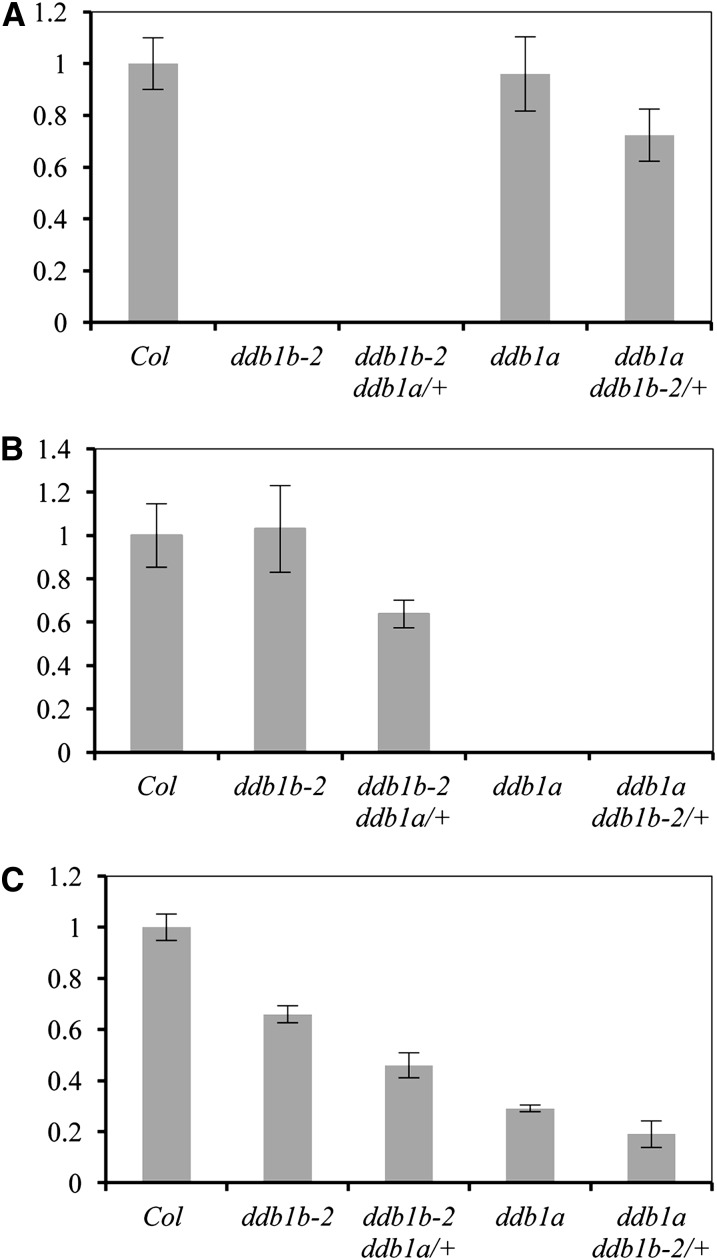
Expression analysis of Arabidopsis *DDB1* alleles. (A) Relative *DDB1B* levels using T-DNA flanking primers P3 and P4. (B) Relative *DDB1A* levels using primers P7 and P8 3′ of T-DNA insertion site. (C) Relative levels of total full-length *DDB1* (both *DDB1A* and *DDB1B)* using common primers C1 and C2. (A-C) Data normalized with *Actin*. Error bars = ± SE (n = 4). Note the segregating *ddb1b-2 ddb1a/*+ and *ddb1a ddb1b-2/*+ consist of pooled populations (2/3 +/− and 1/3 +/+).

Arabidopsis DDB1A and DDB1B are 91% identical at the amino acid level and are both expressed throughout plant development. *ddb1a*-null alleles exhibit no obvious developmental defects, suggesting that *DDB1B* acts redundantly during normal development. However, *ddb1b* null alleles appear lethal as viable homozygotes cannot be obtained (Schroeder *et al.* 2002; Al Khateeb and Schroeder 2007). In this study we use a T-DNA allele in the *DDB1B* gene, *ddb1b-2* (SALK_061944), previously described by Bernhardt *et al.* (2010). This T-DNA insertion results in truncation of the last 112 amino acids of DDB1B, preventing interaction with DDB2, and presumably other WD40 proteins, but not CUL4 (Bernhardt *et al.* 2010), thus results in a partial loss of function allele (Figure 1A). To examine the role of total DDB1 activity in Arabidopsis growth and development, we combined *ddb1b-2* with a previously described *ddb1a* mutation (Schroeder *et al.* 2002). This *ddb1a* allele also retains transcript 5′ of the T-DNA insertion at the beginning of the tenth exon (data not shown), but prevents accumulation of the full length *DDB1A* transcript (Figure 1B). However, the resulting protein would be only 252 amino acids long (23% of 1088 total amino acids); thus, it would be expected to be a stronger allele than that of *ddb1b-2*. Transcript levels of both *DDB1A* and *DDB1B* are unchanged in the *ddb1b-2* and *ddb1a* mutant backgrounds respectively (Figure 1, A and B).

As previously described (Bernhardt *et al.* 2010; Dumbliauskas *et al.* 2011), although single *ddb1a* and *ddb1b-2* mutants exhibit no obvious developmental phenotypes, the *ddb1a ddb1b-2* double mutant is embryo lethal, preventing analysis of traits later in development. Therefore, we used the two single mutants and the two segregating heterozygotes (*ddb1a ddb1b-2/+* and *ddb1b-2 ddb1a/+*) to examine the effect of *DDB1* dose on development and abiotic stress responses. The respective heterozygotes exhibit decreased levels of *DDB1B* and *DDB1A* transcripts [note the segregating *ddb1b-2 ddb1a/+* and *ddb1a ddb1b-2/+* consist of pooled populations (2/3 +/− and 1/3 +/+)] (Figure 1, A and B). Primers in conserved regions of the *DDB1* genes reveal that total full-length *DDB1* transcript level decreases from wild type to *ddb1b-2* to *ddb1b-2 ddb1a/+* to *ddb1a* to *ddb1a ddb1b-2/+* (Figure 1C), consistent with data that *DDB1A* transcript levels are approximately twice those of *DDB1B* (Al Khateeb and Schroeder 2007).

DDB1 complexes have been implicated in photomorphogenesis and other light-regulated processes, so the phenotypes of *ddb1a*, *ddb1b-2*, and the *ddb1a ddb1b-2/+* and *ddb1b-2 ddb1a/+* heterozygotes in dark-grown seedlings, light-grown seedlings, and adults were examined. In dark-grown seedlings, no hypocotyl length or apical hook phenotypes were observed (Supporting Information, Figure S1). Similarly, in light-grown conditions, no phenotypes with respect to hypocotyl length, cotyledon width, anthocyanin or chlorophyll content were observed (Figure S2). In adults, no effects on flowering time (days and leaves), height, silique length, or apical dominance were detected, although a slight increase in rosette diameter was observed in the *ddb1b-2 ddb1a/+* heterozygotes (Figure S3). Thus, in our hands, a single wild-type copy of either *DDB1A* or *DDB1B* is sufficient for most development.

**Figure 2:**
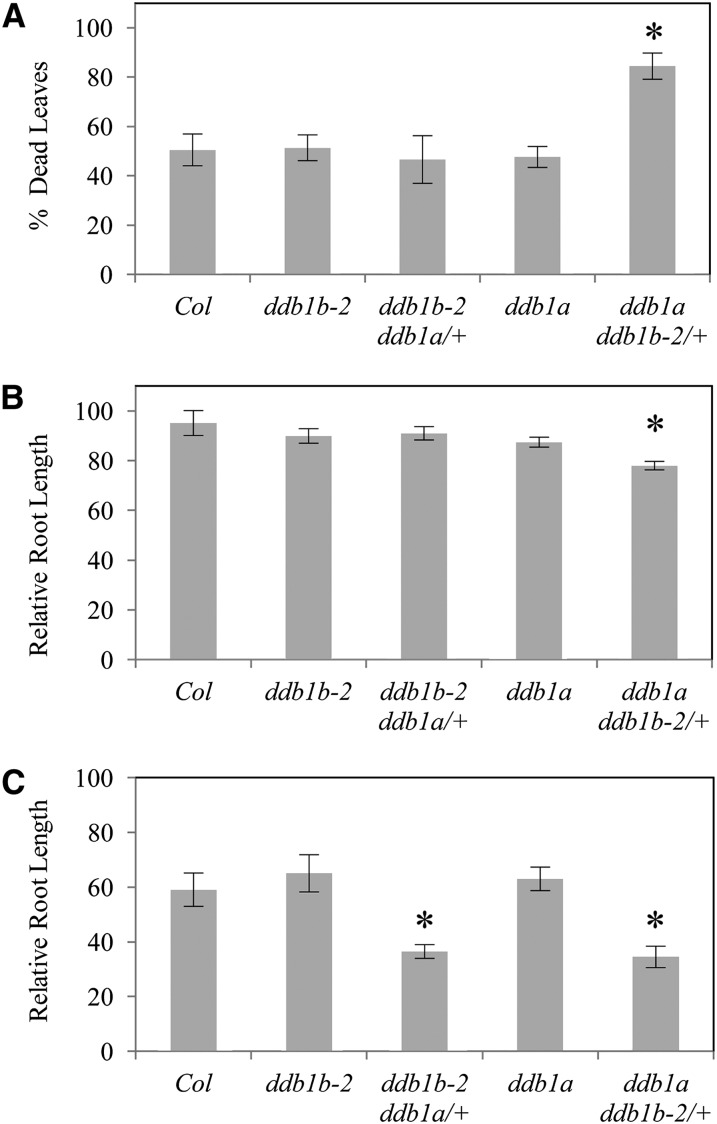
UV tolerance in *ddb1b-2* and *ddb1a* mutant backgrounds. (A) Percent dead leaves in shoots of adult plants after irradiation with 450 Jm^-2^ UV-C and 3 d dark incubation (n = 12). (B) Relative root length (%) of seedlings exposed to 600 Jm^-2^ UV-C then incubated in long-day conditions for 2 d. (C) Relative root length (%) of seedlings exposed to 1500 Jm^-2^ UV-C then incubated under dark conditions for 3 d. For (B) and (C), root length is relative to unirradiated controls of the same genotype (n = 25). Error bars indicate SE; and *P ≤ 0.05 of single mutants relative to Col or *ddb1a ddb1b-2/*+ and *ddb1b-2 ddb1a/*+ relative to *ddb1a* and *ddb1b-2* respectively. Note the segregating *ddb1b-2 ddb1a/*+ and *ddb1a ddb1b-2/*+ in the above experiments consists of a pooled population (2/3 +/− and 1/3 +/+).

In several systems, DDB1 has been shown to interact with the WD40 proteins DDB2 and CSA during GG-NER and TC-NER respectively to repair UV-damaged DNA (Ganpudi and Schroeder 2011). Here we examine UV sensitivity in *ddb1a*, *ddb1b-2*, and the segregating *ddb1a ddb1b-2/+* and *ddb1b-2 ddb1a/+* heterozygotes to determine the roles of *DDB1A* and *DDB1B* in Arabidopsis UV tolerance. Adult plants were exposed to UV-C and leaf damage was scored (Figure 2A). There was no significant difference in percentage dead leaves between the single mutants *ddb1b-2* and *ddb1a* and wild type. Similarly, no differences between *ddb1b-2 ddb1a/+* and *ddb1b-2* were observed, but *ddb1a ddb1b-2/+* exhibited greater levels of tissue death than *ddb1a*. In seedlings, as in adult plants, the single mutants and *ddb1b-2 ddb1a/+* did not exhibit sensitive phenotypes; however, *ddb1a ddb1b-2/+* again exhibited a root UV-sensitive phenotype 2 d after UV irradiation when incubated in long-day conditions (Figure 2B). However, increased UV sensitivity in both *ddb1b-2 ddb1a/+* and *ddb1a ddb1b-2/+* was observed after treatment with a higher dose of UV and 3-d dark incubation (Figure 2C). Note these experiments used segregating *ddb1b-2 ddb1a/+* and *ddb1a ddb1b-2/+* populations (2/3 +/− and 1/3 +/+) and thus may underestimate the phenotype of the heterozygotes.

**Figure 3:**
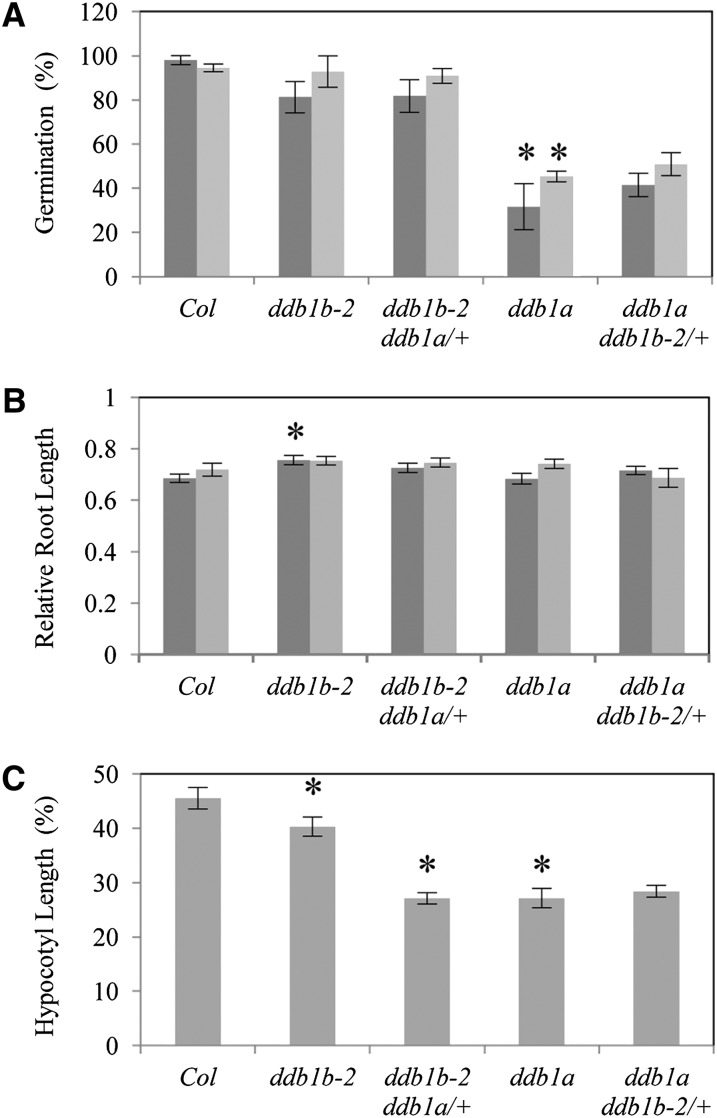
Abiotic stress response in *ddb1b-2* and *ddb1a* mutant backgrounds. (A) Percent germination on media containing 100 mM NaCl (dark gray bars) or 200 mM Mannitol (light gray bars) 3 d after stratification (n = 2 experimental repeats of 30−50 seeds each). Note 100% germination was observed for all genotypes on control plates after 3 d. (B) Relative root length on vertically aligned plates with 100 mM NaCl (dark gray bars) or 200 mM Mannitol (light gray bars) (n = 10). Root length is relative that of the same genotype on control plates. (C) Relative hypocotyl length (%) of 4-day-old dark-grown seedlings treated with 45°C for 4 hr, then dark-grown at 20° for an additional 4 d. Hypocotyl length is relative to untreated controls of the same genotype (n = 15). For A-C, error bars indicate SE and *P ≤ 0.05 of single mutants relative to Col, or *ddb1a ddb1b-2/*+ and *ddb1b-2 ddb1a/*+ relative to *ddb1a* and *ddb1b-2* respectively. Note the *ddb1b-2 ddb1a/*+ and *ddb1a ddb1b-2/*+ in the above experiments consists of a pooled segregating population (2/3 +/− and 1/3 +/+).

The DCAF proteins DWA1, DWA2 and DWA3 have recently been implicated in ABA signaling and NaCl tolerance (Lee *et al.* 2010, 2011); thus, we examined the contributions of *DDB1A* and *DDB1B* to salt and osmotic stress tolerance using germination assays. Although *ddb1b-2* and *ddb1b-2 ddb1a/+* exhibited normal germination rates on both 100 mM NaCl and 200 mM Mannitol, *ddb1a* and *ddb1a ddb1b-2/+* exhibited reduced germination rates in both these conditions (Figure 3A). Thus, *DDB1A* appears to have a critical role in regulation of germination during stress conditions, whereas no significant effect of *DDB1B* mutation could be detected in either the wild type or *ddb1a* background. Although *ddb1a* and *ddb1a ddb1b-2/+* exhibited delayed germination, they did not exhibit any root growth phenotypes after 7 d in these conditions (Figure 3B). In fact, root growth in *ddb1b-2* was found be slightly resistant to salt. Finally, we examined the role of *DDB1A* and *DDB1B* in heat sensitivity by analyzing the effect of heat on dark-grown hypocotyl length (Figure 3C). *ddb1b-2* exhibited mild heat sensitivity while *ddb1b-2 ddb1a/+*, *ddb1a* and *ddb1a ddb1b-2/+* all exhibited similar strong sensitivity. Thus both *DDB1A* and *DDB1B* contribute to heat tolerance.

### Interactions between *ddb1b* and *det1*

DET1, a master repressor of photomorphogenesis, interacts both biochemically and genetically with DDB1A (Schroeder *et al.* 2002). Here we examine genetic interactions between *det1* and *ddb1b-2* in dark-grown seedlings, light-grown seedlings and adults.

**Figure 4:**
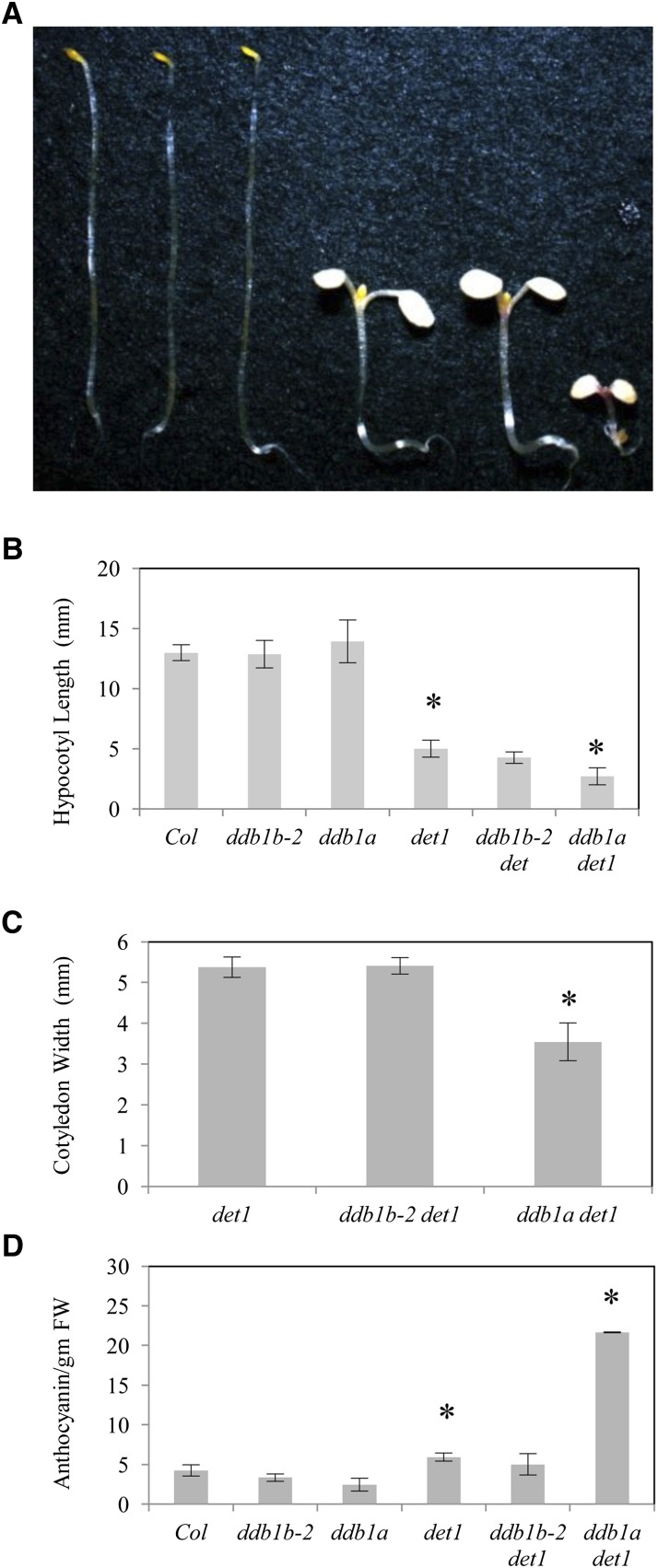
*ddb1b-2 det1* dark-grown seedlings. (A) From left: Col, *ddb1b-2*, *ddb1a*, *det1*, *ddb1b-2 det1*, and *ddb1a det1*. (B) Hypocotyl length (n = 15). (C) Cotyledon width (n = 15). (D) Anthocyanin content (A_530_- A_657_ / g fresh weight) (n = 2). Error bars indicate 95% CI; *P ≤ 0.05 of single mutants relative to Col or of double mutants relative to *det1*.

In dark-grown seedlings, *det1* mutants exhibit a constitutively de-etiolated phenotype with short hypocotyls, open cotyledons, and increased anthocyanin content (Chory *et al.* 1989). As described previously (Schroeder *et al.* 2002), in the dark *ddb1a det1* mutants exhibit decreased hypocotyl length and cotyledon width as well as increased anthocyanin content relative to *det1* single mutants (Figure 4A). However the *ddb1b-2 det1* double mutants did not significantly differ from *det1* with respect to any of these phenotypes. Thus, *ddb1a* has a stronger effect on *det1* phenotypes in the dark than *ddb1b-2*.

**Figure 5:**
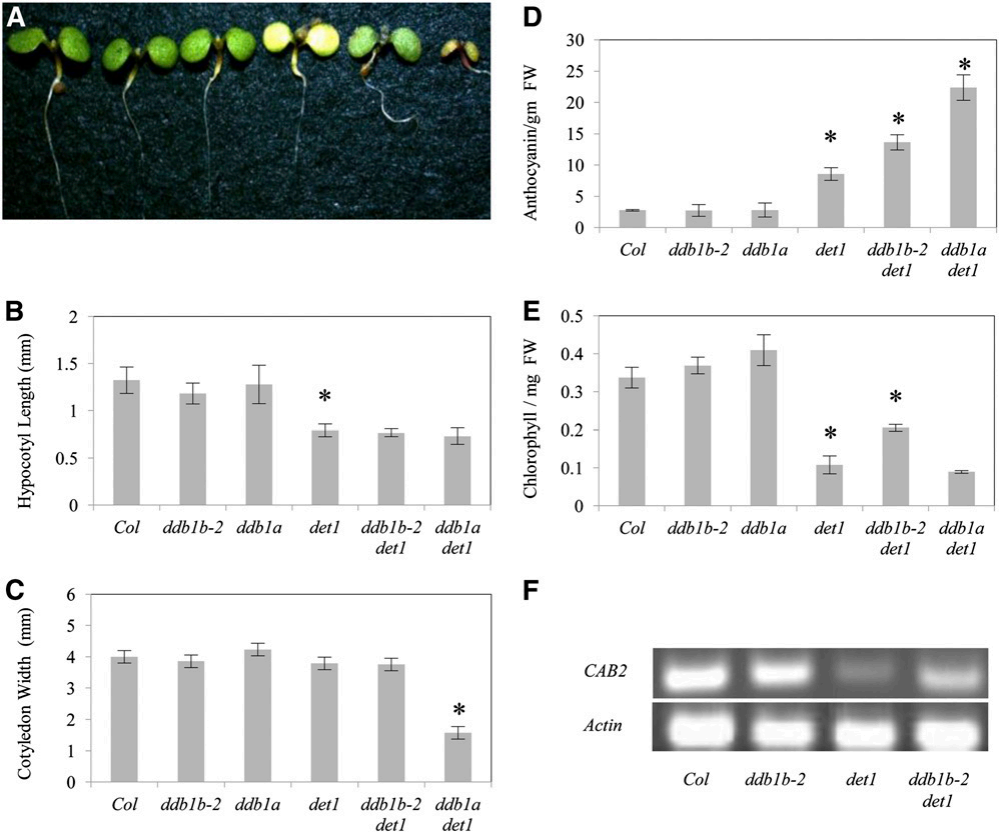
*ddb1b-2 det1* light-grown seedlings. (A) From left: Col, *ddb1b-2*, *ddb1a*, *det1*, *ddb1b-2 det1*, and *ddb1a det1*. (B) Hypocotyl length (n = 15). (C) Cotyledon width (n = 15). (D) Anthocyanin content (A_530_- A_657_ / g fresh weight) (n = 2). (E) Chlorophyll content (µg of chlorophyll / mg of fresh weight) (n = 2). Error bars indicate 95% CI; *P ≤ 0.05 of single mutants relative to Col or of double mutants relative to *det1*. (F) Semiquantitative RT-PCR analysis of *Chlorophyll A/B – Binding Protein 2 (CAB2)* At1g29920 expression levels in Col, *ddb1b-2*, *det1* and *ddb1b-2 det1*.

In light-grown seedlings, *det1* mutants are small with decreased chlorophyll and increased anthocyanin levels. In the light as in the dark, the *ddb1a det1* mutants exhibit decreased cotyledon width and increased anthocyanin levels relative to *det1* (Figure 5). Although the *ddb1b-2 det1* mutants did not differ from *det1* with respect to hypocotyl length or cotyledon width, they did however exhibit enhanced anthocyanin levels, intermediate between those of *det1* and *ddb1a det1* (Figure 5D). Interestingly, *ddb1b-2 det1* mutants exhibited higher chlorophyll levels than *det1*, thus *ddb1b-2* partially suppresses the *det1* pale phenotype (Figure 5E). This suppression appears to occur at the transcriptional level, since *CAB2* transcript levels are higher in *ddb1b-2 det1* than in *det1* (Figure 5F).

**Figure 6:**
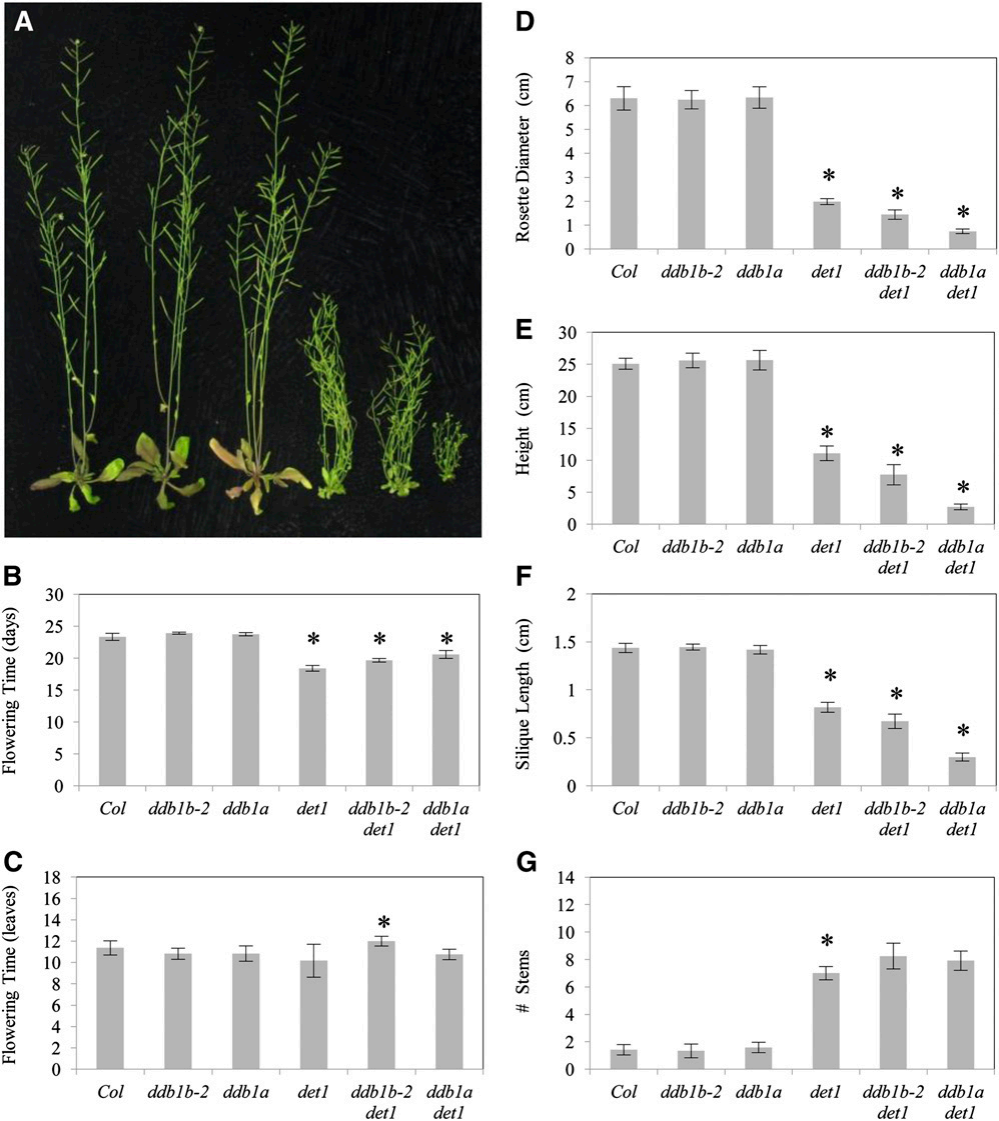
*ddb1b-2 det1* adult phenotypes. (A) From left: Col, *ddb1b-2*, *ddb1a*, *det1*, *ddb1b-2 det1*, and *ddb1a det1*. (B) Flowering time (in days). (C) Flowering time (in leaves). (D) Rosette diameter. (E) Plant height. (F) Silique length. (G) Number of stems. For (B−G), error bars indicate 95% CI (n = 12); *P ≤ 0.05 of single mutants relative to Col or of double mutant relative to *det1*.

We also compared the effect of *ddb1a* and *ddb1b-2* on *det1* phenotypes in adult plants (Figure 6). *det1* mutants exhibit early flowering (Pepper and Chory 1997). Flower bud emergence in *det1* occurs at approximately 18 d in long-day conditions in contrast to wild-type plants, where bud emergence occurs at approximately 24 d. Like *ddb1a det1* (bud emergence at approximately 22 d), *ddb1b-2 det1* double mutants partially suppress early flowering in *det1*, with bud emergence at approximately 20 d under long-day conditions (Figure 6B). *ddb1b-2* also partially suppressed *det1* early flowering in short-day conditions (Figure S4A). In terms of leaf number at flowering, *ddb1b-2 det1* double mutants flowered at significantly increased leaf number relative to *det1* in long day (Figure 6B); however, no effect was observed in short-day conditions (Figure S4B).

*det1* adults are dwarf in stature, with reduced rosette diameter, height, and silique length relative to wild type (Figure 6, D−F). All three of these parameters are further decreased in *ddb1a det1* double mutants (62%, 75%, and 64% smaller, respectively); thus, *ddb1a* strongly enhances the *det1* dwarf phenotype. *ddb1b-2* also enhanced these three *det1* phenotypes, but to a lesser extent than *ddb1a* (27%, 30% and 17% respectively). In addition, *ddb1b-2* enhanced these three *det1* phenotypes in short-day conditions as well (Figure S4, C−E). *det1* also has decreased apical dominance resulting in increased inflorescence number. *ddb1b-2* does not affect this phenotype in either long-day or short-day conditions (Figure 6G and Figure S4F).

### Interaction between *ddb1b* and *cop1*

**Figure 7:**
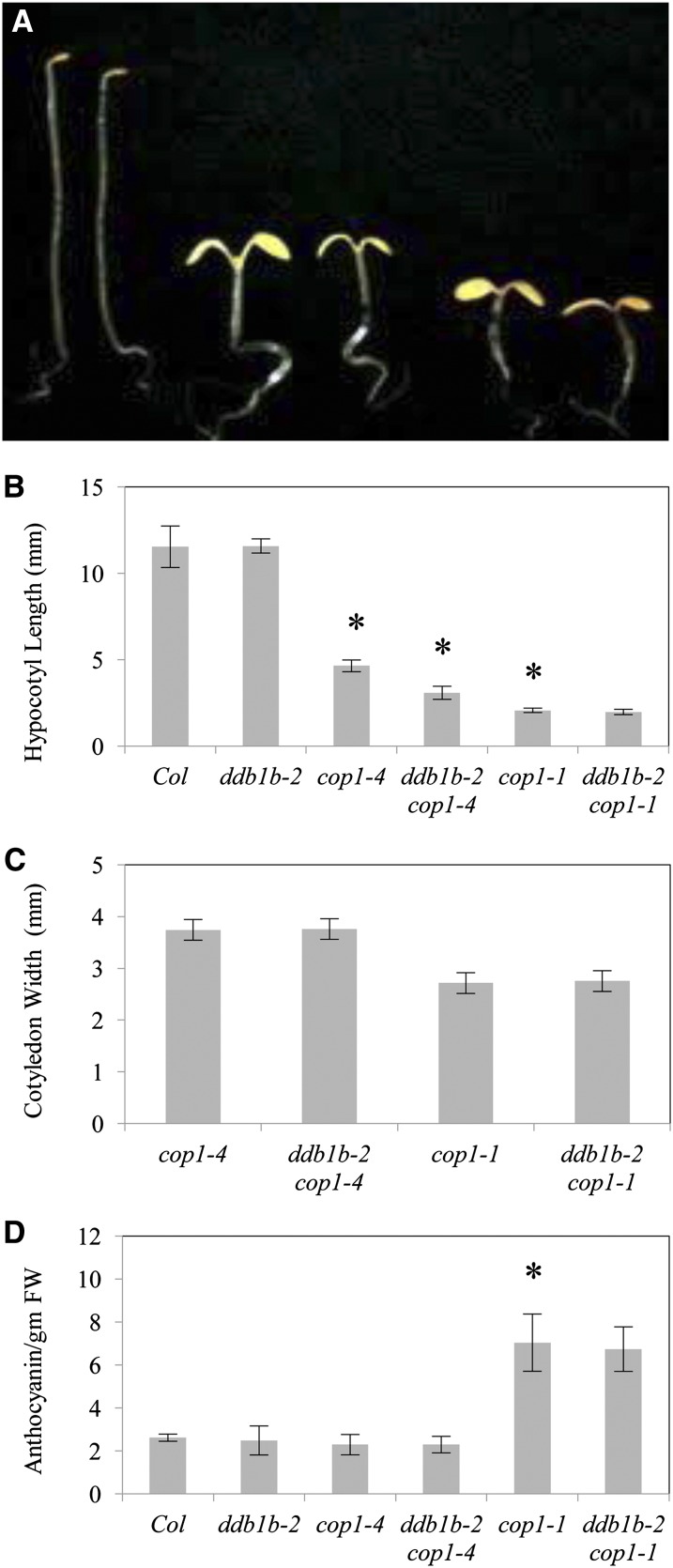
*ddb1b-2 cop1* dark-grown seedlings. (A) From left: Col, *ddb1b-2*, *cop1-4*, *ddb1b-2 cop1-4*, *cop1-1*, and *ddb1b-2 cop1-1*. (B) Hypocotyl length (n = 15). (C) Cotyledon width (n = 15). (D) Anthocyanin content (A_530_- A_657_ / g fresh weight) (n = 2). Error bars indicate 95% CI; *P ≤ 0.05 of single mutants relative to Col or of *ddb1b-2 cop1-4* and *ddb1b-2 cop1-1* relative to *cop1-4* and *cop1-1*, respectively.

**Figure 8:**
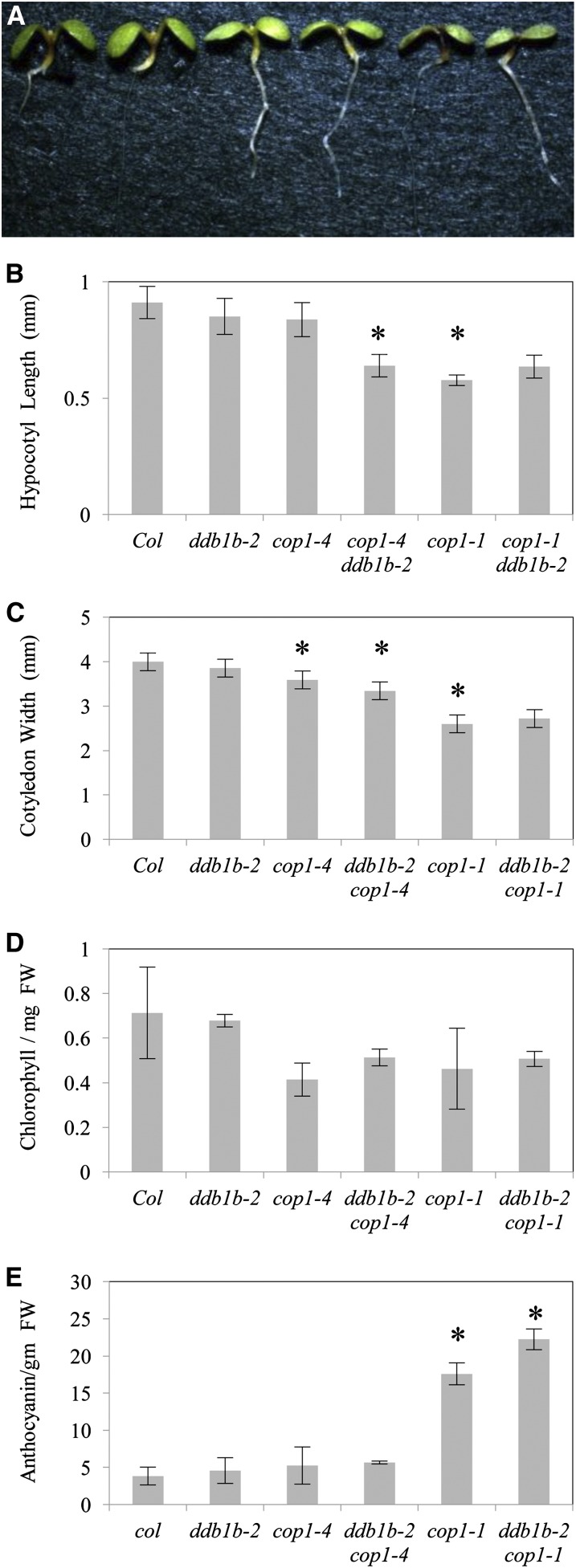
*ddb1b-2 cop1* light-grown seedlings. (A) From left: Col, *ddb1b-2*, *cop1-4*, *ddb1b-2 cop1-4*, *cop1-1*, and *ddb1b-2 cop1-1*. (B) Hypocotyl length (n = 15). (C) Cotyledon width (n = 15). (D) Anthocyanin content (A_530_- A_657_ / g fresh weight) (n = 2). (E) Chlorophyll content (µg of chlorophyll / mg of fresh weight) (n = 2). Error bars indicate 95% CI; *P ≤ 0.05 of single mutants relative to Col or of *ddb1b-2 cop1-4* and *ddb1b-2 cop1-1* relative to *cop1-4* and *cop1-1*, respectively.

**Figure 9:**
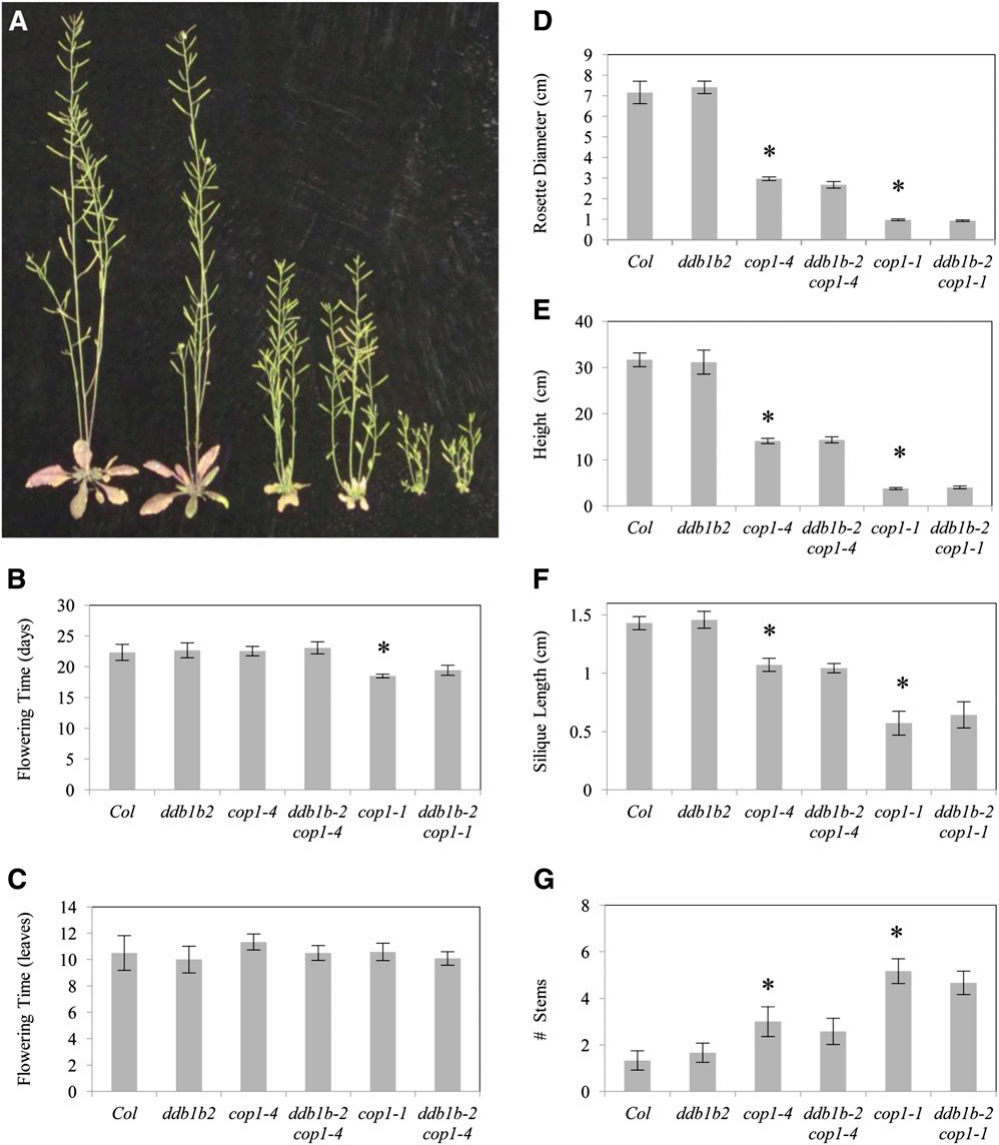
*ddb1b-2 cop1* adult phenotypes. (A) From left: Col, *ddb1b-2*, *cop1-4*, *ddb1b-2 cop1-4*, *cop1-1*, and *ddb1b-2 cop1-1*. (B) Flowering time (in days). (C) Flowering time (in leaves). (D) Rosette diameter. (E) Plant height. (F) Silique length. (G) Number of stems. For B-G, error bars indicate 95% CI (n = 12); *P ≤ 0.05 of single mutants relative to Col or of *ddb1b-2 cop1-4* and *ddb1b-2 cop1-1* relative to *cop1-4* and *cop1-1*, respectively.

Photomorphogenic protein COP1 has also been shown to form a CUL4-DDB1 complex via interactions between DDB1 and the WDXR motif in the COP1 WD40 domain (Chen *et al.* 2010). Here we examine *ddb1b-2 cop1* genetic interactions during development. *ddb1b-2* double mutants were generated with two *cop1* alleles: a strong allele (*cop1-1*, internal deletion potentially altering the conformation of the WD40 domain) and a relatively weak allele (*cop1-4*, truncated protein lacking the WD40 domain) (McNellis *et al.* 1994). *cop1* mutants, like *det1*, exhibit a constitutively photomorphogenic phenotype in the dark (Figure 7). In dark-grown seedlings, *ddb1b-2* enhanced the short hypocotyl phenotype in *cop1-4* but not *cop1-1* (Figure 7B). No differences in cotyledon width or anthocyanin content were observed in either *ddb1b-2 cop1-4* or *ddb1b-2 cop1-1* double mutants relative to their respective single mutants (Figure 7, C and D). In light-grown seedlings (Figure 8), *ddb1b-2* decreased both hypocotyl length and cotyledon width in *cop1-4* but not *cop1-1* (Figure 8, B and C). *ddb1b-2* had no significant effect on chlorophyll content in either *cop1* allele (Figure 8D). With respect to anthocyanin content, *ddb1b-2* had no effect on *cop1-4* but enhanced anthocyanin levels in *cop1-1* (Figure 8E). In adults, *ddb1b-2* did not significantly alter any *cop1* phenotype in either long-day or short-day conditions (Figure 9, Figure S4). Thus, genetic interactions between *ddb1b-2* and *cop1* appear to be developmentally regulated.

Thus *ddb1b-2* had no effect on *cop1* adult phenotypes but significantly affected *det1* adult phenotypes. In contrast, in 7-d-old dark-grown seedlings, *ddb1b-2* enhanced the *cop1-4* short hypocotyl phenotype but did not significantly affect *det1* phenotypes. To examine the effect of seedling age on dark phenotypes, seedlings were grown for 5 or 6 d in the dark and hypocotyl length and cotyledon width measured. After 5 and 6 d, *ddb1b-2* now significantly enhanced the *det1* short hypocotyl phenotype, as well as that of *cop1-4*, but not *cop1-1* (Figure S5A). Nonetheless the effect of *ddb1b-2* on hypocotyl length was consistently stronger in the *cop1-4* background, resulting in 30 and 25% reduction in hypocotyl length in 6 and 5 d, respectively, than in the *det1* background, where the double mutants were 16 and 13% shorter than the *det1* single mutants at 6 and 5 d, respectively. No significant effect of *ddb1b-2* on cotyledon width was observed in these conditions (Figure S5B).

## Discussion

The purpose of this study was to identify differences between *DDB1A* and *DDB1B* in terms of redundant and distinct functions, and to examine genetic interactions with specific DDB1 interactors. Interestingly, whereas our analysis of *ddb1a* and *ddb1b-2* single mutants and segregating heterozygotes detected no phenotypes except for increased rosette diameter in *ddb1b-2 ddb1a/+*, using the same viable *ddb1b-2* allele (SALK_069144), Bernhardt *et al.* (2010) identified a variety of developmental phenotypes in these lines. These phenotypes included increased dark hypocotyl length in *ddb1a* and *ddb1a ddb1b-2/+*, accelerated flowering in long-day conditions in terms of leaf number in all lines, late flowering in long-day conditions in terms of days in *ddb1b-2 ddb1a/+*, decreased height in *ddb1a ddb1b-2/+*, and decreased silique length in both *ddb1a ddb1b-2/+* and *ddb1b-2 ddb1a/+*. Pazhouhandeh *et al.* (2011) observed no flowering phenotypes in long-day conditions with the *ddb1b* SALK_069144 allele but did detect early flowering in long-day conditions with respect to both days and leaves with *ddb1a*. The differences between studies could be due to differences in the *ddb1a* allele used or experimental conditions.

In UV tolerance assays, we did not detect any sensitivity in *ddb1b-2* single mutants. Similar results were obtained using the same allele in UV-B and UV-C tolerance assays by Bernhardt *et al.* (2010) and Castells *et al.* (2011), respectively. In adult UV tolerance assays, only *ddb1a ddb1b-2/+* exhibited UV sensitivity. Similarly, in seedlings treated with UV followed by light incubation, sensitivity was also observed only in *ddb1a ddb1b-2/+*. Thus in these two assays only the lines with the lowest total *DDB1* transcript levels (Figure 1C) exhibited sensitivity.

In light conditions, both photolyase enzymes and NER contribute to repair of UV-damaged DNA. In dark conditions, however, plants are dependent on NER for repair. In our experiments, with dark incubation post irradiation both *ddb1a ddb1b-2/+* and *ddb1b-2 ddb1a/+* are sensitive to UV treatment. These experiments also used a stronger UV dose than the light assay, 1500 Jm^-2^ compared with 600 Jm^-2^. Thus, when the demand for NER is amplified by increasing the amount of UV damage and by removing the contribution of photolyases, neither a single wild-type copy of *DDB1B* nor *DDB1A* is sufficient for wild-type levels of UV tolerance. However, two wild-type alleles of either gene are sufficient.

Recently, Lee *et al.* (2010, 2011) characterized WD40 proteins involved in ABA signaling (DWA1, DWA2, and DWA3). The single and double *dwa* mutants are sensitive to ABA and NaCl, as are *CUL4* co-suppression lines. Although DWA1, 2, and 3 all interact with both DDB1A and DDB1B *in vitro*, we find that germination in *ddb1a* is more sensitive to 100 mM NaCl and 200 mM Mannitol than germination in *ddb1b-2* or wild type. Interestingly, the sensitivity of *ddb1a* in germination had no effect on root growth, suggesting that *DDB1A* and *DDB1B* act redundantly to regulate this phenotype.

Heat sensitivity was observed in *ddb1b-2*, *ddb1a*, *ddb1b-2 ddb1a/+*, and *ddb1a ddb1b-2/+*, with plants lacking one or both copies of *DDB1A* the most sensitive. Other recent studies in our lab also implicate *DDB1A* in heat response (V. Ly, A. Hatherell, E. Kim, and D. Schroeder, unpublished data). Consistent with a role for *DDB1A* in heat tolerance, AtGenExpress data (Kilian *et al.* 2007) indicates that heat elevates *DDB1A* transcript levels, but not *DDB1B* levels. In contrast, neither mannitol nor salt treatment strongly induce *DDB1A* levels, but *DDB1B* levels decrease (Figure S6).

DET1 interacts biochemically with CUL4-DDB1 and exhibits genetic interactions with both *CUL4* and *DDB1A* (Schroeder *et al.* 2002; Chen *et al.* 2006). Although *ddb1a* enhances all *det1* phenotypes in dark-grown seedlings, *ddb1b-2* only weakly enhances the short hypocotyl phenotype. In light-grown seedlings, *ddb1b-2* enhanced the *det1* high anthocyanin phenotype and suppressed the *det1* low chlorophyll phenotype. This suppression appears to occur at the transcriptional level since *CAB2* transcript levels are also increased in the *ddb1b-2 det1* double mutant. The fact that the *ddb1b-2 det1* double mutant suppresses the *det1* chlorophyll phenotype suggests the two proteins are not acting together in this instance, and that this suppression may be indirect, for example via another complex. In previous studies, *ddb2* was also found to suppress the *det1* low chlorophyll phenotype (Al Khateeb and Schroeder 2007). DET1 has recently been shown to act as a transcriptional repressor (Lau *et al.* 2011), but this does not explain the underexpression of *CAB2* in *det1* in light, thus DET1 regulation of *CAB2* in the light may be indirect. It is not clear whether DDB1 is involved in regulation of transcription by DET1 (Lau *et al.* 2011). *CAB2* promoter analysis has shown that a HY5-binding element is required for DET1 light-regulation of *CAB2*, and *hy5* mutants suppress the *det1* pale phenotype (Maxwell *et al.* 2003).

In adult plants, *ddb1a* enhances the *det1* small phenotype, resulting in decreased rosette diameter, height and silique length, and partially suppresses early flowering in *det1* in terms of days. For all these phenotypes *ddb1b-2* has a similar effect on *det1* as *ddb1a*, but to a lesser extent. Given that our *ddb1a* allele is potentially stronger than the *ddb1b-2* partial loss of function allele, and that *DDB1A* is expressed at greater levels than *DDB1B* throughout development (Al Khateeb and Schroeder 2007; Bernhardt *et al.* 2010), these results are consistent with both DDB1A and DDB1B contributing to DET1 regulation of adult growth.

Only a few effects of *ddb1b-2* on *cop1* phenotypes were observed. *ddb1b-2* enhanced the short hypocotyl phenotype in both dark and light-grown *cop1-4*. Because *ddb1b-2* had smaller and no effects on *det1* dark and light-grown hypocotyl length, respectively, DDB1B appears to be more critical for COP1 function than for DET1 function with respect to regulation of hypocotyl length. In light-grown seedlings, *ddb1b-2* enhanced anthocyanin levels in *cop1-1* and *det1*, suggesting that DDB1B has a common role in regulation of light anthocyanin levels. In adults, *ddb1b-2* had no effect on any phenotypes in either *cop1* allele. In contrast, *ddb1b-2* modified the majority of *det1* adult phenotypes, indicating that in adults DDB1B is more critical for DET1 function than for COP1 function. Thus, the requirement for DDB1B seems to vary in the course of development, from COP1-specific interactions in hypocotyls to DET1-specific in adults. Whether this specificity is due to differential levels, cellular localization, or biochemical interactions of DDB1B *vs.* DDB1A is unknown. *In vitro* COP1 interacts with both GST-DDB1B and GST-DDB1A, and FLAG-DDB1B coimmunoprecipitates both DET1 and COP1 from light and dark-grown seedlings (Chen *et al.* 2010). In onion cells, GFP fusions of both DDB1A and DDB1B are localized in both the nucleus and cytoplasm, though a larger proportion of DDB1B is cytoplasmic (Zhang *et al.* 2008). COP1 is predominantly nuclear in the dark and cytoplasmic in the light, whereas DET1 is exclusively nuclear (Lau and Deng 2012). In addition, the COP1-4 and COP1-1 forms of the COP1 protein exhibit defects in nuclear localization (Stacey *et al.* 1999, 2000; Nakagawa and Komeda 2004). Thus perhaps cytoplasmic colocalization is the basis of *ddb1b cop1* interaction in seedlings, but requires further analysis. The *cop1* alleles used in this study, *cop1-4* (truncated protein predicted to lack the WD40 domain) and *cop1-1* (internal deletion potentially altering the conformation of the WD40 domain) (McNellis *et al.* 1994), would be predicted to be compromised in their ability to interact with DDB1 proteins as well as other proteins such as photoreceptors and transcription factors that interact with the COP1 WD40 domain (Yi and Deng 2005). Thus, any *ddb1b-2 cop1* genetic interactions observed may be indirect.

Thus in this study we have examined the relative contributions of DDB1B and DDB1A to stress response, as well as DET1 and COP1 function, and find that there appears to be developmental regulation of DDB1 interactions.

## Supplementary Material

Supporting Information
