# Supplementary material for: Genetic Interactions of Arabidopsis thaliana Damaged DNA Binding Protein 1B (DDB1B) With DDB1A, DET1, and COP1
Source: G3 (Bethesda). 2013 Mar 1;3(3):493–503. doi: 10.1534/g3.112.005249 (PMC3583456; doi:10.1534/g3.112.005249)
Supplement: Supporting Information [file supp_3_3_493__index.html]

Supporting Information 

# Genetic Interactions of *Arabidopsis thaliana Damaged DNA Binding Protein 1B* (*DDB1B*) With *DDB1A*, *DET1*, and *COP1*

## Supporting Information for Ganpudi and Schroeder, 2013

**Files in this Data Supplement:**

- Supporting Information - Figures S1-S6 (PDF, 488 KB)
- Figure S1 - *ddb1b-2* and *ddb1a* dark-grown seedlings (PDF, 92 KB)
- Figure S2 - *ddb1b-2* and *ddb1a* light-grown seedlings (PDF, 100 KB)
- Figure S3 - *ddb1b-2* and *ddb1a* adult phenotypes (PDF, 152 KB)
- Figure S4 - *ddb1b-2 det1* and *ddb1b-2 cop1* adult growth parameters under short day conditions (PDF, 127 KB)
- Figure S5 - *ddb1b-2 det1* and *ddb1b-2 cop1* six (dark grey bars) and five (light grey bars) day old dark grown seedling phenotypes. (PDF, 100 KB)
- Figure S6 - Effect of abiotic stress on relative expression levels of *DDB1A* (blue) and *DDB1B* (red) (PDF, 187 KB)
