# Supplementary material for: Genetic Interactions of Arabidopsis thaliana Damaged DNA Binding Protein 1B (DDB1B) With DDB1A, DET1, and COP1
Source: G3 (Bethesda). 2013 Mar 1;3(3):493–503. doi: 10.1534/g3.112.005249 (PMC3583456; doi:10.1534/g3.112.005249)
Supplement: Supporting Information [file supp_3.3.493_005249SI.pdf]

G3 Genes | Genomes | Genetics

Genetic Interactions of *Arabidopsis thaliana Damaged DNA Binding Protein 1B* (*DDB1B)* with *DDB1A, DET1* and *COP1*

Ashwin L. Ganpudi[1] and Dana F. Schroeder

Department of Biological Sciences, University of Manitoba, Winnipeg, MB, Canada, R3T 2N2
[1] Present address: Dupont Knowledge Centre, Hyderabad- 500078, Andhra Pradesh, India

Corresponding author:
Dana F. Schroeder
Department of Biological Sciences
University of Manitoba
Winnipeg, MB, Canada
R3T 2N2
Phone: (204) 474-7106
Fax: (204) 474-7588
Email: schroed3@cc.umanitoba.ca

DOI: 10.1534/g3.112.005249

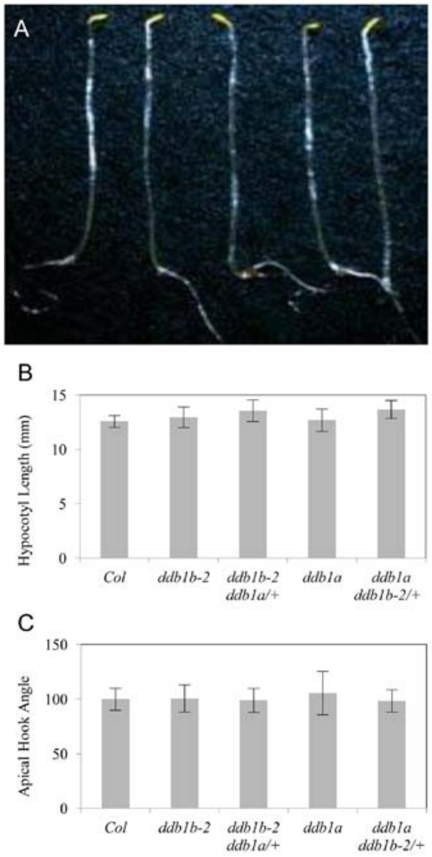

Figure S1 *ddb1b-2* and *ddb1a* dark-grown seedlings. (A) from left: Col, *ddb1b-2, ddb1b-2 ddb1a/+, ddb1a* and *ddb1a ddb1b-2/+*. (B) Hypocotyl length (n=15). (C) Apical Hook Angle (n=15). Error bars indicate 95% CI. Single mutants relative to Col and *ddb1a ddb1b-2/+* and *ddb1b-2 ddb1a/+* relative to *ddb1a* and *ddb1b-2* respectively exhibited no significant differences. Note the segregating *ddb1b-2 ddb1a/+* and *ddb1a ddb1b-2/+* in the above experiments consists of a pooled population (2/3 +/- and 1/3 +/+).

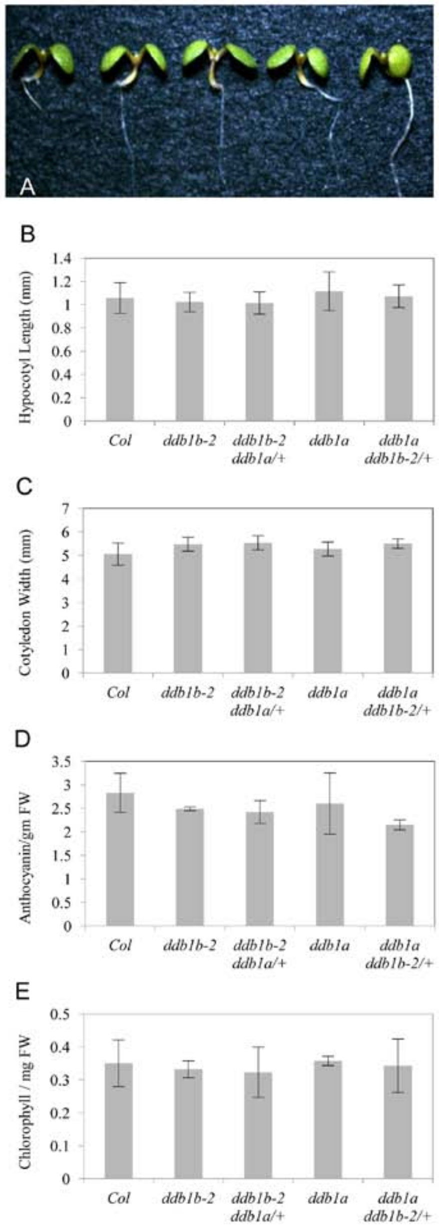

Figure S2 *ddb1b-2* and *ddb1a* light-grown seedlings. (A) from left: Col, *ddb1b-2, ddb1b-2 ddb1a/+, ddb1a* and *ddb1a ddb1b-2/+*. (B) Hypocotyl length (n=15). (C) Apical Hook Angle (n=15). (D) Anthocyanin content (A_{530}- A_{657} / g fresh weight) (n=2). (E) Chlorophyll content (µg chlorophyll / mg fresh weight) (n=2). Error bars indicate 95% CI. Single mutants relative to Col and *ddb1a ddb1b-2/+* and *ddb1b-2 ddb1a/+* relative to *ddb1a* and *ddb1b-2* respectively exhibited no significant differences. Note the segregating *ddb1b-2 ddb1a/+* and *ddb1a ddb1b-2/+* in the above experiments consists of a pooled population (2/3 +/- and 1/3 +/+).

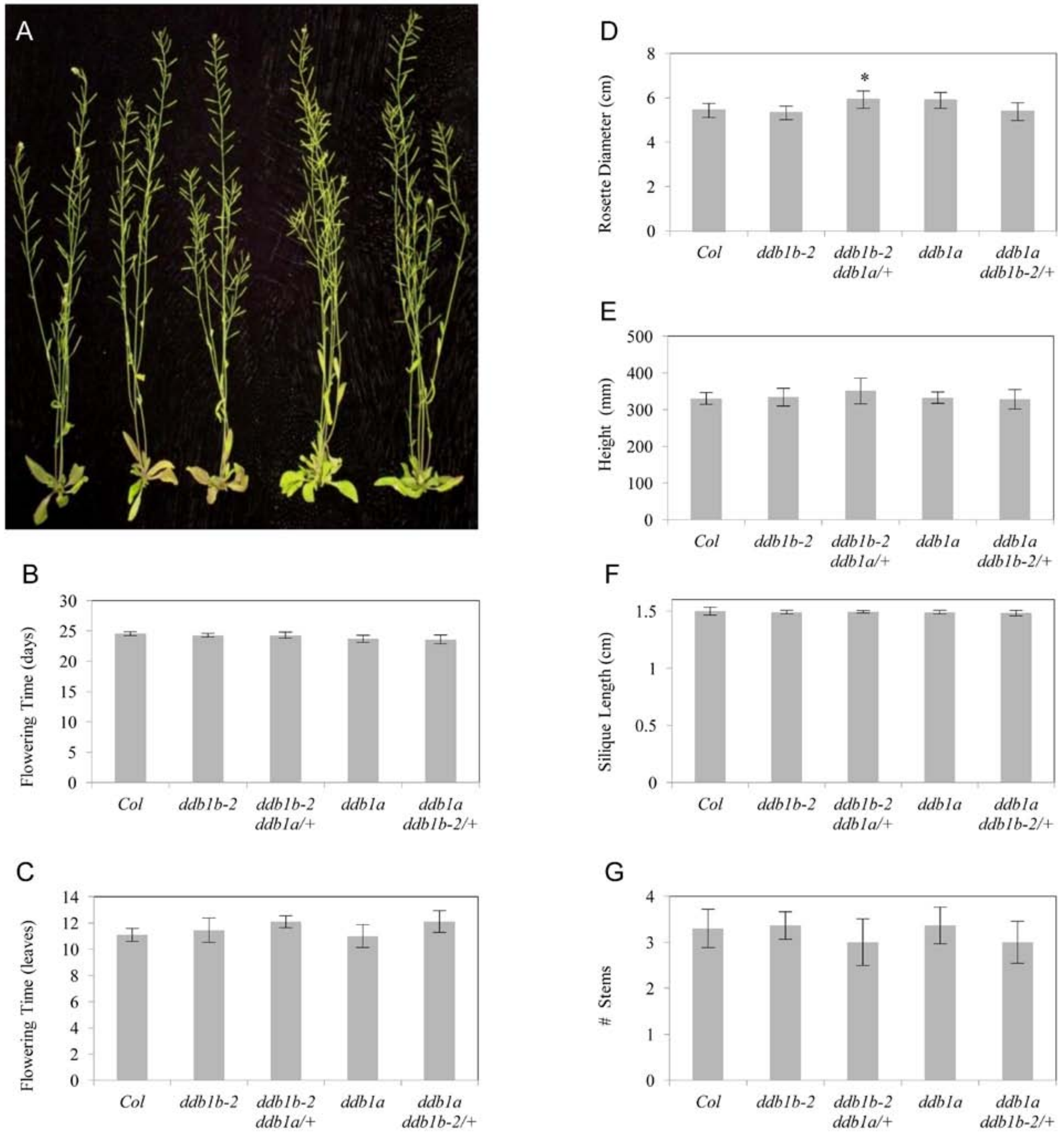

Figure S3 *ddb1b-2* and *ddb1a* adult phenotypes. (A) from left: Col, *ddb1b-2, ddb1a, ddb1b-2 ddb1a/+,* and *ddb1a ddb1b-2/+*. (B) Flowering time (in days). (C) Flowering time (in leaves). (D) Rosette Diameter. (E) Plant height. (F) Silique length. (G) Number of stems. Error bars indicate 95% CI (n=12) and * indicates P< 0.05 of single mutants relative to Col and *ddb1a ddb1b-2/+* and *ddb1b-2 ddb1a/+* relative to *ddb1a* and *ddb1b-2* respectively. Note only confirmed *ddb1b-2 ddb1a/+* and *ddb1ba ddb1b-2/+* were used here. Abnormal ovule distribution was used as a phenotypic marker.

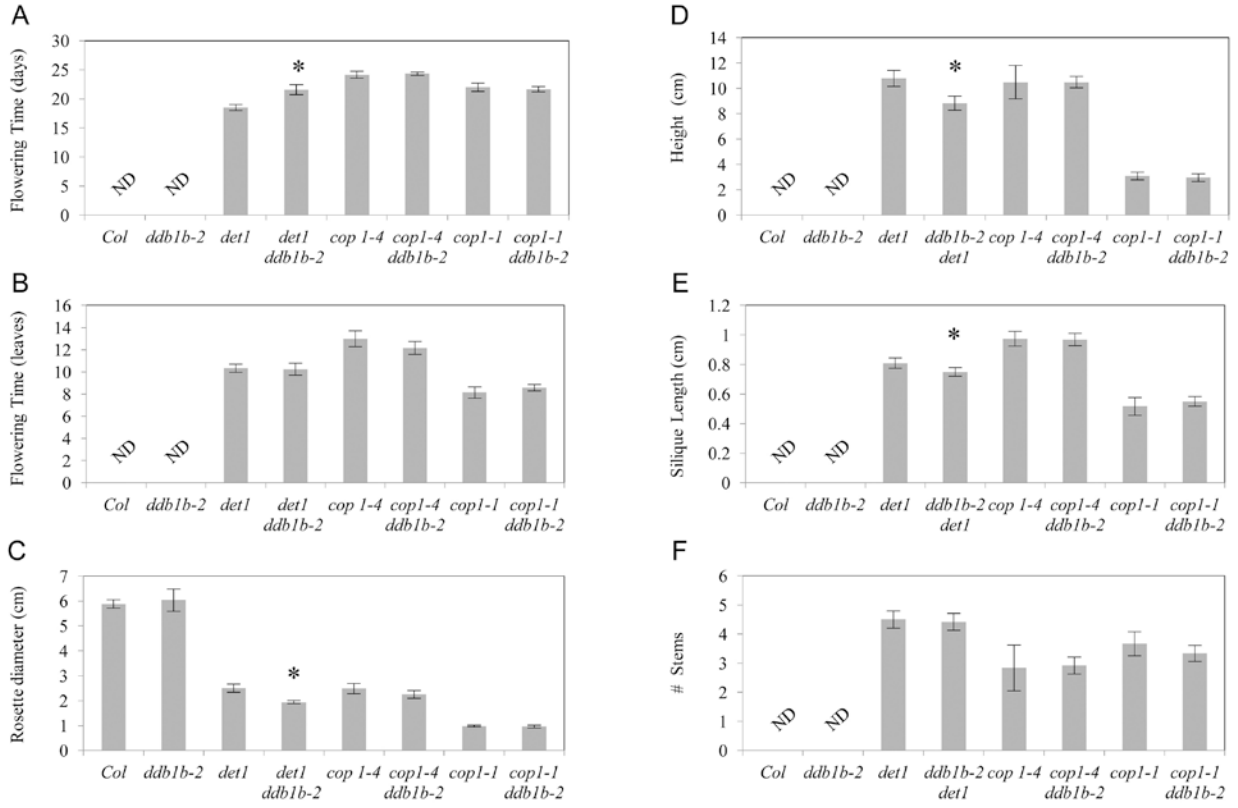

Figure S4 *ddb1b-2 det1* and *ddb1b-2 cop1* adult growth parameters under short day conditions. (A) Flowering time (in days). (B) Flowering time (in leaves). (C) Rosette Diameter. (D) Plant height. (E) Silique length. (F) Number of stems. Error bars indicate 95% CI (n=12) and * indicates $P \leq 0.05$ of double mutants relative to their respective single mutants. ND = not determined

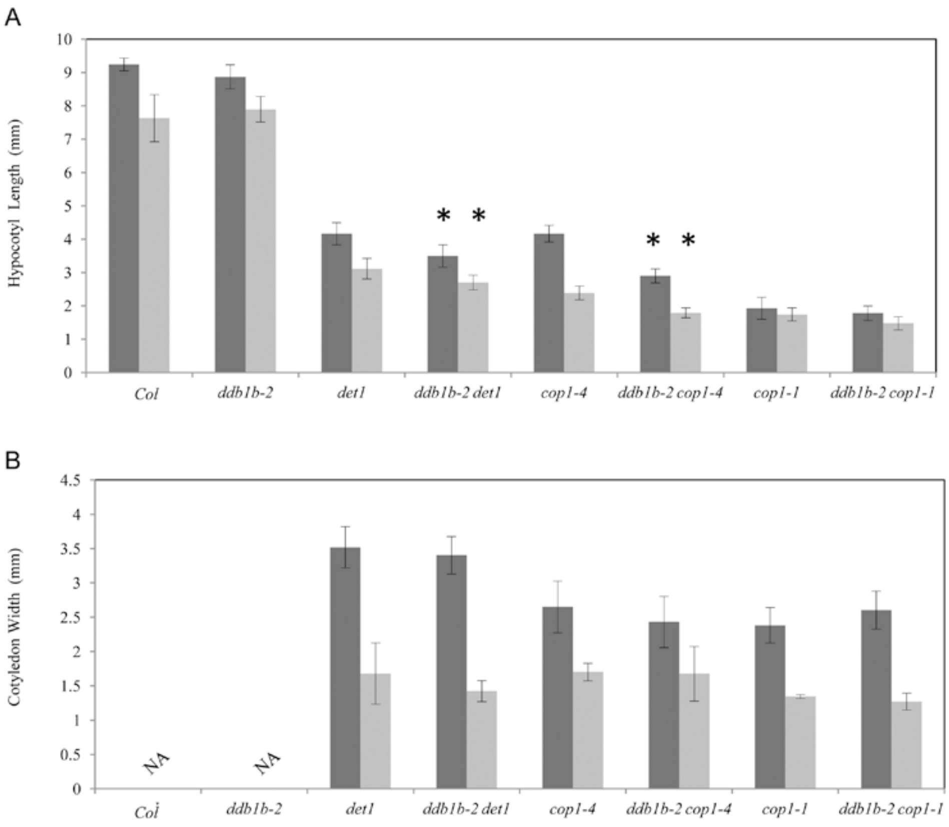

Figure S5 *ddb1b-2 det1* and *ddb1b-2 cop1* six (dark grey bars) and five (light grey bars) day old dark grown seedling phenotypes. (A) Hypocotyl length. (B) Cotyledon width. Error bars indicate 95% CI (n=10) and * indicates P≤ 0.05 of double mutants relative to their respective single mutants. NA = not applicable

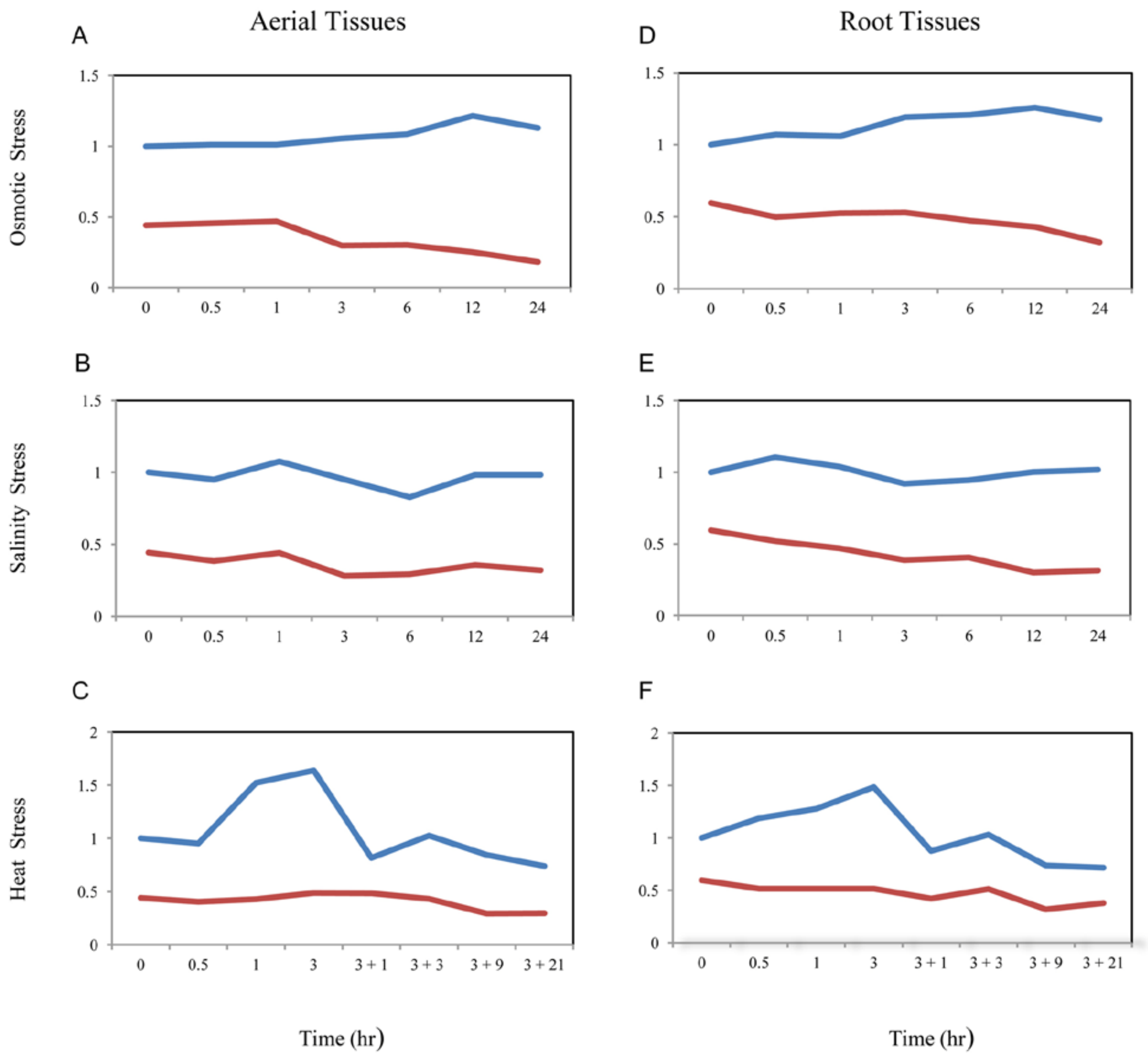

Figure S6 Effect of abiotic stress on relative expression levels of *DDB1A* (blue) and *DDB1B* (red). Expression relative to *DDB1A* control levels in (A-C) aerial tissues and (D-F) roots. (A, D) Osmotic stress (300 mM Mannitol); (B, E) Salt stress (150 mM NaCl) (C, F) Heat stress (38° for 3 hr followed by recovery at 25°). Data from Kilian *et al.* (2007) accessed via AtGenExpress
