# Supplementary material for: Genetic Interactions of Arabidopsis thaliana Damaged DNA Binding Protein 1B (DDB1B) With DDB1A, DET1, and COP1
Source: G3 (Bethesda). 2013 Mar 1;3(3):493–503. doi: 10.1534/g3.112.005249 (PMC3583456; doi:10.1534/g3.112.005249)
Supplement: Supporting Information [file supp_3.3.493_FigureS1.pdf]

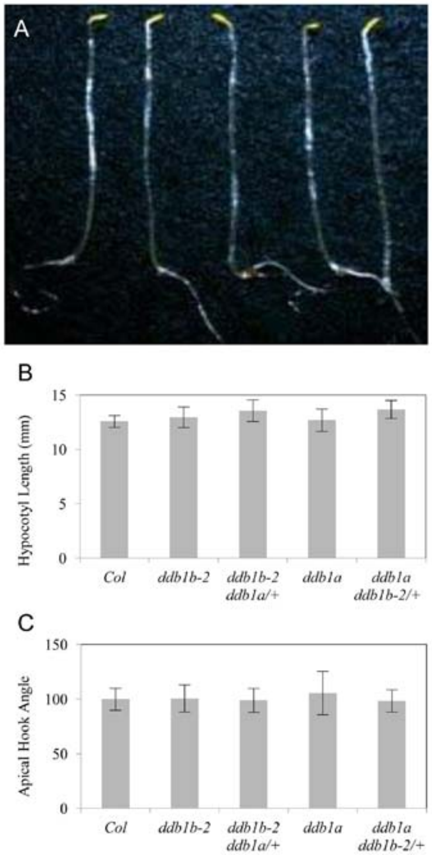

Figure S1 *ddb1b-2* and *ddb1a* dark-grown seedlings. (A) from left: Col, *ddb1b-2, ddb1b-2 ddb1a/+, ddb1a* and *ddb1a ddb1b-2/+*. (B) Hypocotyl length (n=15). (C) Apical Hook Angle (n=15). Error bars indicate 95% CI. Single mutants relative to Col and *ddb1a ddb1b-2/+* and *ddb1b-2 ddb1a/+* relative to *ddb1a* and *ddb1b-2* respectively exhibited no significant differences. Note the segregating *ddb1b-2 ddb1a/+* and *ddb1a ddb1b-2/+* in the above experiments consists of a pooled population (2/3 +/- and 1/3 +/+).
