# Supplementary material for: Genetic Interactions of Arabidopsis thaliana Damaged DNA Binding Protein 1B (DDB1B) With DDB1A, DET1, and COP1
Source: G3 (Bethesda). 2013 Mar 1;3(3):493–503. doi: 10.1534/g3.112.005249 (PMC3583456; doi:10.1534/g3.112.005249)
Supplement: Supporting Information [file supp_3.3.493_FigureS3.pdf]

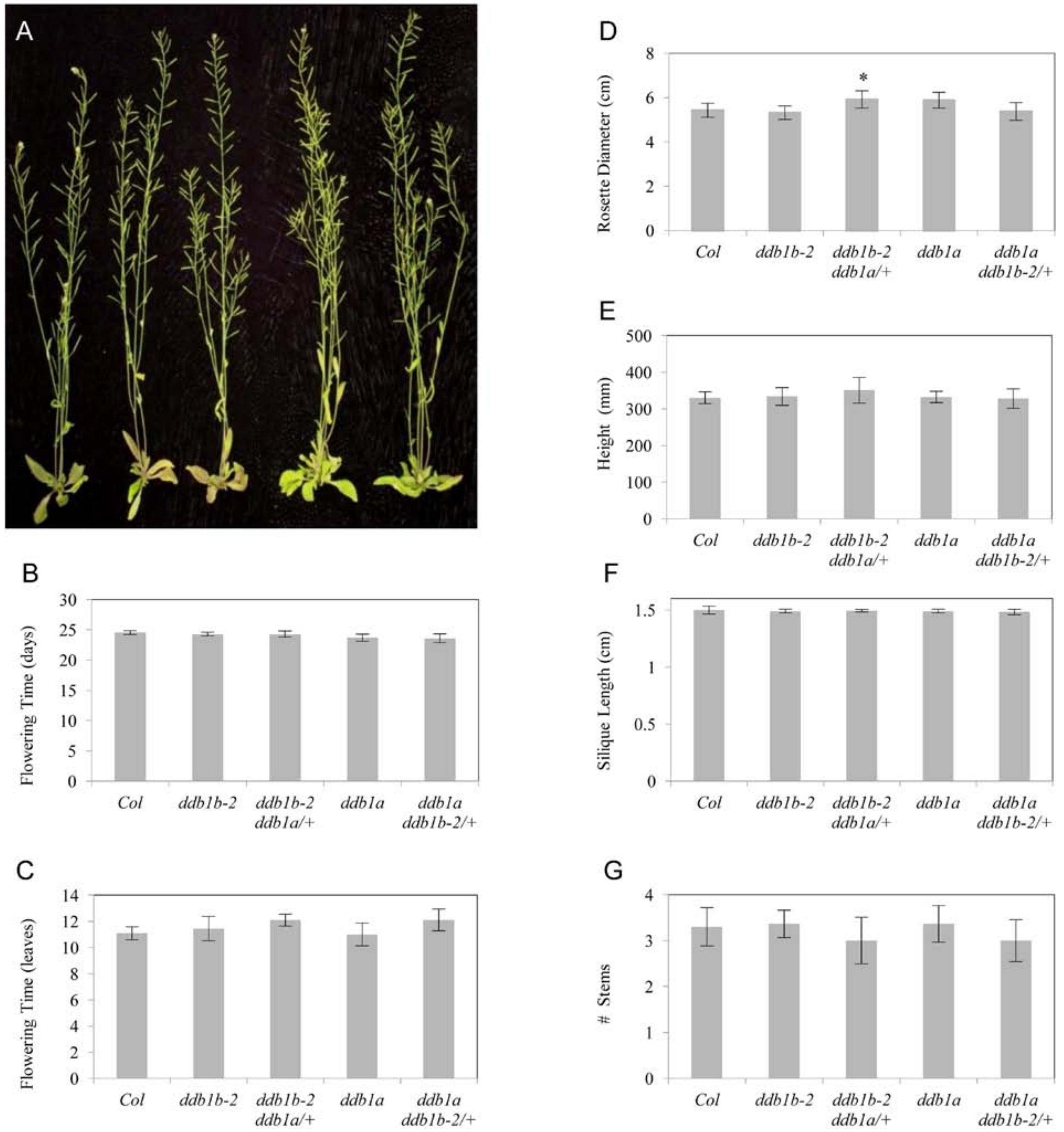

Figure S3 *ddb1b-2* and *ddb1a* adult phenotypes. (A) from left: Col*, ddb1b-2, ddb1a, ddb1b-2 ddb1a/+,* and *ddb1a ddb1b-2/+*. (B) Flowering time (in days). (C) Flowering time (in leaves). (D) Rosette Diameter. (E) Plant height. (F) Silique length. (G) Number of stems. Error bars indicate 95% CI (n=12) and * indicates P< 0.05 of single mutants relative to Col and *ddb1a ddb1b-2/+* and *ddb1b-2 ddb1a/+* relative to *ddb1a* and *ddb1b-2* respectively. Note only confirmed *ddb1b-2 ddb1a/+* and *ddb1ba ddb1b-2/+* were used here*.* Abnormal ovule distribution was used as a phenotypic marker.
