# Supplementary material for: Genetic Interactions of Arabidopsis thaliana Damaged DNA Binding Protein 1B (DDB1B) With DDB1A, DET1, and COP1
Source: G3 (Bethesda). 2013 Mar 1;3(3):493–503. doi: 10.1534/g3.112.005249 (PMC3583456; doi:10.1534/g3.112.005249)
Supplement: Supporting Information [file supp_3.3.493_FigureS4.pdf]

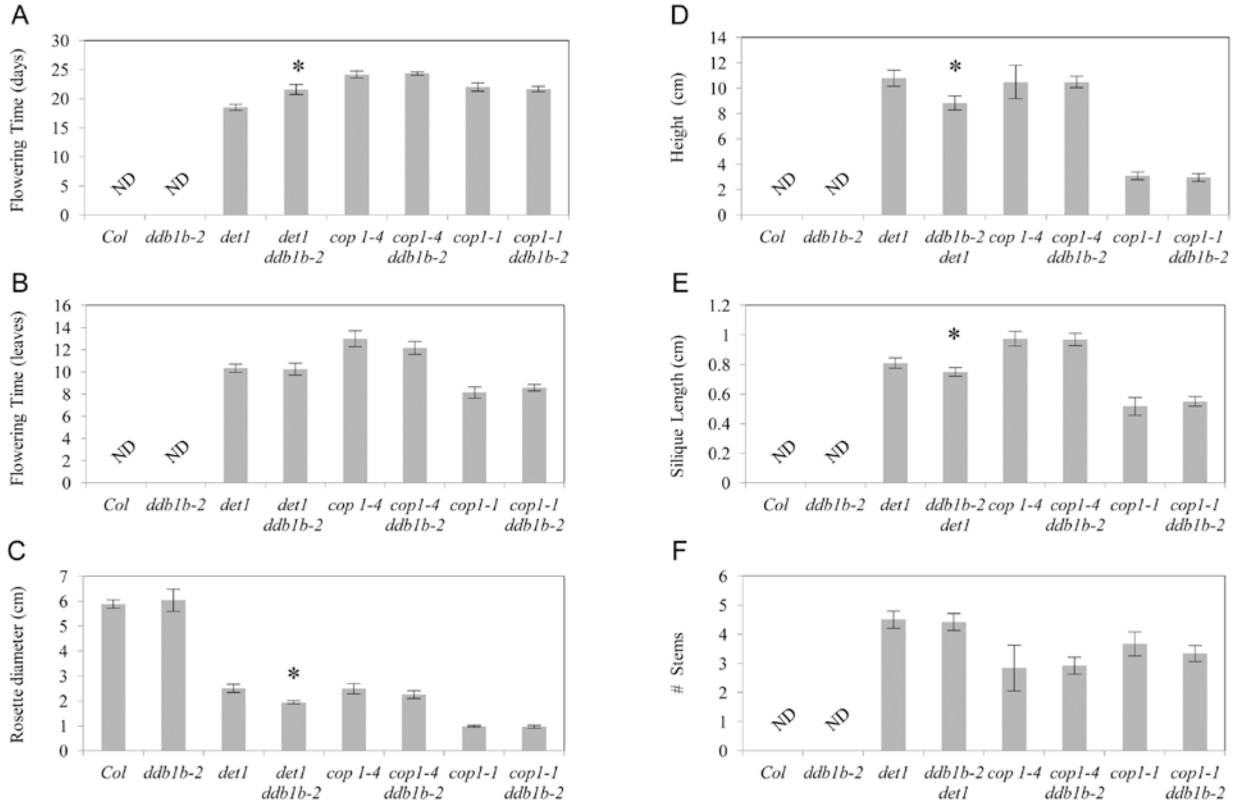


**Figure S4** *ddb1b-2 det1* and *ddb1b-2 cop1* adult growth parameters under short day conditions. (A) Flowering time (in days). (B) Flowering time (in leaves). (C) Rosette Diameter. (D) Plant height. (E) Silique length. (F) Number of stems. Error bars indicate 95% CI (n=12) and * indicates $P \leq 0.05$ of double mutants relative to their respective single mutants. ND = not determined
