# Supplementary material for: Genetic Interactions of Arabidopsis thaliana Damaged DNA Binding Protein 1B (DDB1B) With DDB1A, DET1, and COP1
Source: G3 (Bethesda). 2013 Mar 1;3(3):493–503. doi: 10.1534/g3.112.005249 (PMC3583456; doi:10.1534/g3.112.005249)
Supplement: Supporting Information [file supp_3.3.493_FigureS5.pdf]

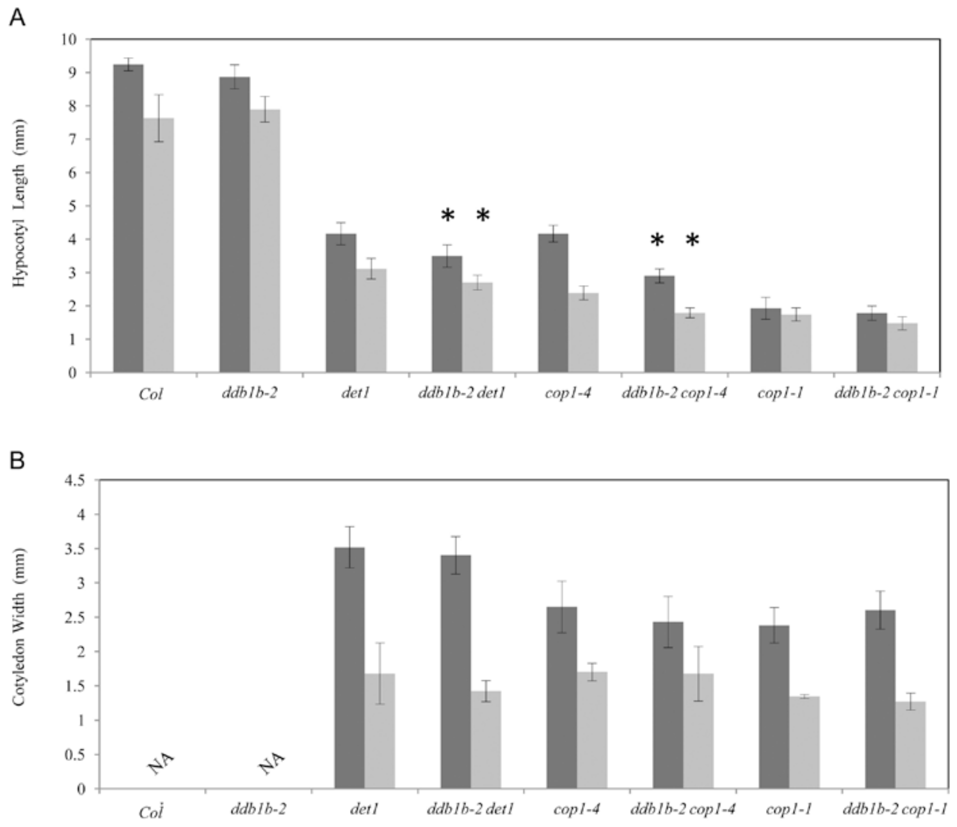

Figure S5 *ddb1b-2 det1* and *ddb1b-2 cop1* six (dark grey bars) and five (light grey bars) day old dark grown seedling phenotypes. (A) Hypocotyl length. (B) Cotyledon width. Error bars indicate 95% CI (n=10) and * indicates $P \leq 0.05$ of double mutants relative to their respective single mutants. NA = not applicable
