# Supplementary material for: Genetic Interactions of Arabidopsis thaliana Damaged DNA Binding Protein 1B (DDB1B) With DDB1A, DET1, and COP1
Source: G3 (Bethesda). 2013 Mar 1;3(3):493–503. doi: 10.1534/g3.112.005249 (PMC3583456; doi:10.1534/g3.112.005249)
Supplement: Supporting Information [file supp_3.3.493_FigureS6.pdf]

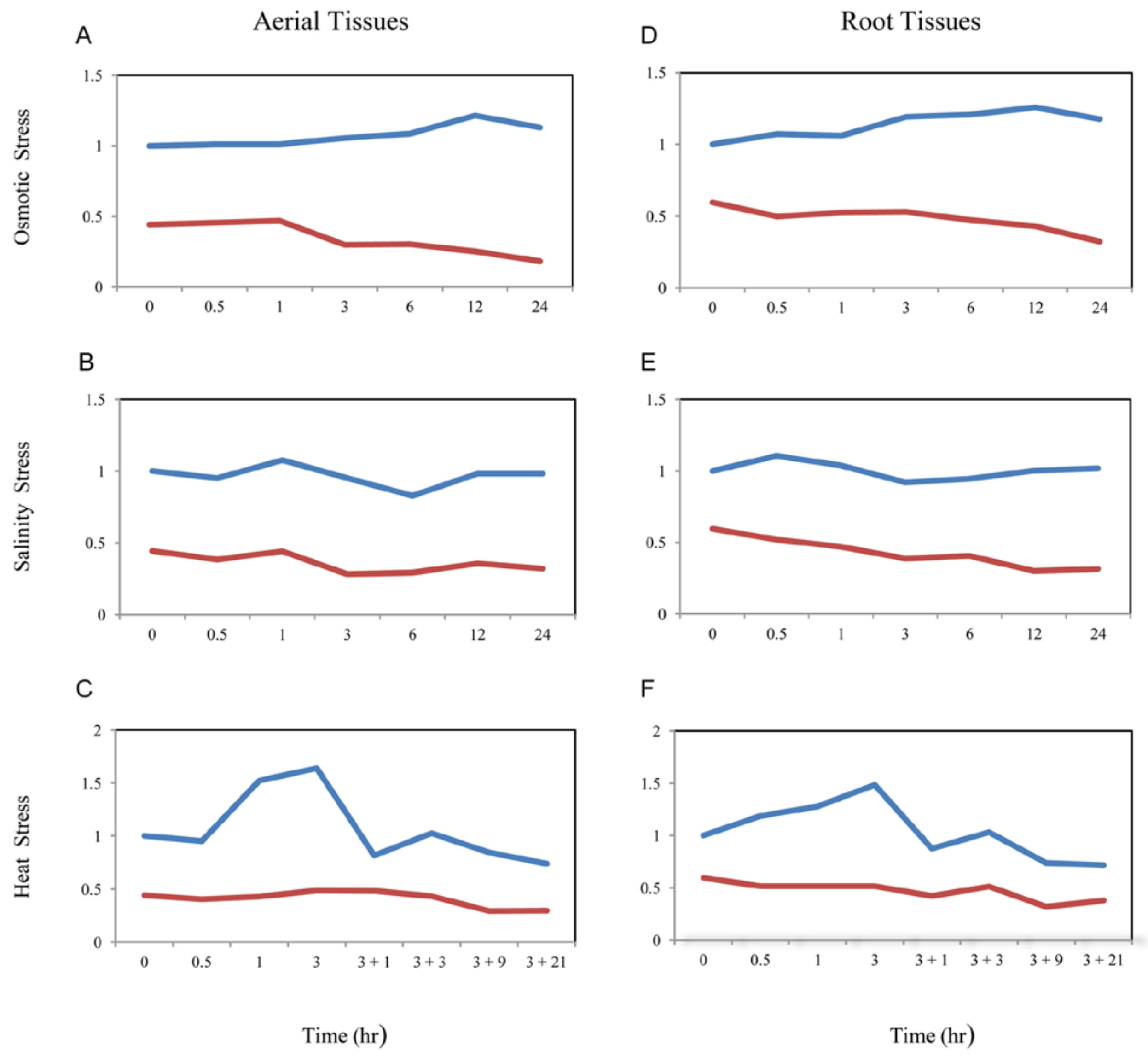

Figure S6 Effect of abiotic stress on relative expression levels of *DDB1A* (blue) and *DDB1B* (red). Expression relative to *DDB1A* control levels in (A-C) aerial tissues and (D-F) roots. (A, D) Osmotic stress (300 mM Mannitol); (B, E) Salt stress (150 mM NaCl) (C, F) Heat stress (38° for 3 hr followed by recovery at 25°). Data from Kilian *et al.* (2007) accessed via AtGenExpress
